# Longitudinal Studies of Aging in Sub-Saharan Africa: Review, Limitations, and Recommendations in Preparation of Projected Aging Population

**DOI:** 10.1093/geroni/igae002

**Published:** 2024-01-23

**Authors:** Olayinka Akinrolie, Anthony O Iwuagwu, Michael E Kalu, Daniel Rayner, Oluwagbemiga Oyinlola, Chigozie D Ezulike, Augustine C Okoh, Adebayo O Makanju, Ebere P Ugwuodo, Immaculata A Ugwuja, Makanjuola Osuolale John, Deborah Adeleke, Chukwuenyegom J Egbumike, Ebuka Miracle Anieto, Ijeoma B Anieto, Chiedozie James Alumona, Ogochukwu Kelechi Onyeso, Blessing Ojembe, Chidinma A Omeje, Ernest C Nwachukwu, Ezinne C Ekediegwu, Kelechi M Onyeso, Ademuyiwa Adeboye, Michael Ibekaku, Omobolade Akinrolie, Chukwuebuka P Onyekere

**Affiliations:** Applied Health Sciences, Faculty of Graduate Studies, University of Manitoba, Winnipeg, Manitoba, Canada; School of Social Sciences, University of New South Wales, Sydney, New South Wales, Australia; Department of Social Work, University of Nigeria, Nsukka, Enugu State, Nigeria; School of Kinesiology and Health Science, Faculty of Health, York University, Toronto, Ontario, Canada; Health Research Methods, Evidence, and Impact, McMaster University, Hamilton, Ontario, Canada; School of Social Work, McGill University, Montreal, Quebec, Canada; Medical Social Services Department, University College Hospital, Ibadan, Oyo state, Nigeria; Department of Social Work, University of Nigeria, Nsukka, Enugu State, Nigeria; Department of Social and Behavioural Sciences, City University of Hong Kong, Kowloon, Hong Kong; Health Research Methods, Evidence, and Impact, McMaster University, Hamilton, Ontario, Canada; Interdisciplinary Social Research Program (Aging and Health), Trent University, Peterborough, Ontario, Canada; School of Health and Life Sciences, Glasgow Caledonian University, Glasgow, UK; Department of Gerontology, Faculty of Social Sciences, University of Southampton, Southampton, UK; Faculty of Nursing Sciences, University of Medical Sciences, Ondo, Ondo State, Nigeria; School of Health and Life Sciences, Glasgow Caledonian University, Glasgow, UK; Evangel University, Akaeze, Akaeze, Ebonyi State, Nigeria; School of Health and Life Sciences, Glasgow Caledonian University, Glasgow, UK; Department of Gerontology, Faculty of Social Sciences, University of Southampton, Southampton, UK; Department of Physiotherapy, College of Basic Medical Sciences, Chrisland University, Abeokuta, Ogun State, Nigeria; Faculty of Health Science, University of Lethbridge, Lethbridge, Alberta, Canada; Faculty of Health Science, University of Lethbridge, Lethbridge, Alberta, Canada; Faculty of Social Work, University of Manitoba, Winnipeg, Manitoba, Canada; Physiotherapy Unit, Asaba Specialist Hospital, Asaba, Delta State, Nigeria; School of Physiotherapy, Dalhousie University, Halifax, Nova Scotia, Canada; Department of Medical Rehabilitation (Physiotherapy), Faculty of Health Sciences and Technology, Nnamdi Azikwe University, Nnewi Campus, Anambra, Nigeria; Department of Estate Management, Faculty of Environmental Sciences, University of Nigeria, Nsukka, Enugu State, Nigeria; Department of Medical Rehabilitation, College of Health Sciences, Obafemi Awolowo University, Ile Ife, Osun State, Nigeria; School of Kinesiology and Health Science, Faculty of Health, York University, Toronto, Ontario, Canada; Department of Obstetrics and Gynecology, Health Science Centre, Winnipeg, Manitoba, Canada; Department of Gerontology, Faculty of Social Sciences, University of Southampton, Southampton, UK; Department of Medical Rehabilitation, Faculty of Health Sciences and Technology, University of Nigeria, Nsukka, Enugu State, Nigeria

**Keywords:** HAALSI, Systematic mapping review, WHO-SAGE

## Abstract

**Background and Objectives:**

The United Nations has projected a 218% increase in older people in Sub-Saharan Africa (SSA) between 2019 and 2050, underscoring the need to explore changes that would occur over this time. Longitudinal studies are ideal for studying and proffering solutions to these changes. This review aims to understand the breadth and use of longitudinal studies on aging in the SSA regions, proffering recommendations in preparation for the projected aging population.

**Research Design and Methods:**

This paper is the third of a four-part series paper of a previous systematic mapping review of aging studies in SSA. We updated the search (between 2021 and 2023) and screened the titles/abstracts and full-text articles by a pair of independent reviewers. Data were extracted using a standardized data-charting form, identifying longitudinal studies in SSA.

**Results:**

We identified 193 studies leveraging 24 longitudinal study data sets conducted at 28 unique sites. The World Health Organization’s Study on Global AGEing and Adult Health (WHO-SAGE) (*n* = 59, 30.5%) and Health and Aging in Africa: A Longitudinal Study of an INDEPTH Community in South Africa (HAALSI) (*n* = 51, 26.4%) were the most used longitudinal data sets. Four studies used more than one longitudinal study data set. Eighteen of the longitudinal study data sets were used only in 1–4 studies. Most (*n* = 150, 77.7%) of the studies used a cross-sectional analytical approach.

**Discussion and Implications:**

Longitudinal studies on aging are sparingly being utilized in SSA. Most analyses conducted across the longitudinal data set were cross-sectional, which hindered the understanding of aging changes that occurred over time that could better inform aging policy and interventions. We call for funding bodies, such as WHO-SAGE, to develop funding competitions that focus on conducting longitudinal analyses, such as structural equation modeling, highlighting changes occurring among the aging population in SSA.

**Translational Significance:** Our findings highlighted that the longitudinal aging data sets in Sub-Saharan Africa need to be more utilized or analyzed to highlight the changes that occur in the aging population at the individual or population level. Regions with similar demographics, such as Northern Africa and Western Asia, should explore how longitudinal aging data sets are utilized in their regions. International funding or collaboration on longitudinal aging data set analysis is needed in the Sub-Saharan region in order to understand the projections of an increasingly aging population.

The global population is aging, but it varies across different regions. As of 2019, the regions with the highest number of individuals aged 65 or older are Eastern and Southeastern Asia, with approximately 260.6 million people, and Europe and Northern America, with roughly 200.4 million. Conversely, Sub-Saharan Africa (SSA) has a lower proportion of older adults, with 31.9 million individuals in this age group (Kalu et al., 2021; Velkoff & Kowal, 2006). Nonetheless, the United Nations predicts that the population of older adults in SSA will experience a remarkable 218% increase by 2050, exceeding the current numbers in the regions with the highest population of older adults in 2019 (Kalu et al., 2021; Velkoff & Kowal, 2006). The significant rise in the aging population in SSA has been attributed to increasing life expectancy, improving healthcare, and declining fertility rate in the region (Velkoff & Kowal, 2006). Moreover, because aging is a natural biological phenomenon that cannot be reversed, this rapid population aging, which has been estimated to continue even in the coming decades, could pose several challenges globally as well as in SSA, affecting families and societies (Kalu et al., 2021; Uchenwoke et al., 2020; Velkoff & Kowal, 2006).

The “double-effect” of population aging, which refers to the cumulative impact on the healthcare system and the economy, is a pressing concern in countries with a higher proportion of older adults, such as Japan, Canada, the United States, and the UK (Smith & Longlands, 2010; United Nations, 2019). However, the effect of this phenomenon would be even more devastating in SSA countries, such as Nigeria, Ghana, Kenya, and Uganda, because these countries are projected to have an increased number of older adults, defined as individuals aged 65 years and older (United Nations, 2019). These countries already face significant challenges in the health and social care sector, including poor infrastructure, limited access to medicines, and a shortage of healthcare workers (Harrison, 2003; Kalu et al., 2021; Onen et al., 2019; United Nations, 2019). As the number of older adults increases in these countries, the pressure on these already limited resources will only increase, putting strain on the health and social care systems (Adandom et al., 2020; Kalu et al., 2021; United Nations, 2019).

The significant challenges presented by the rapid growth of aging populations in SSA underscore the pressing need to review the advancements made in aging research in the region. Specifically, placing greater emphasis on longitudinal data could help shed light on the physical, cognitive, and social changes individuals undergo as they age, offering insights into how best to prepare for the region’s aging population. A plethora of literature has conceptualized longitudinal studies in different ways. The most notable consensus definition about longitudinal study is that it employs continuous or repeated approaches to follow a particular individual or group over a period of time, which can either be decades or years (Diggle et al., 2002). Frison and Pocock (1992) viewed longitudinal studies as a general observational approach using qualitative or quantitative data, collected on any combination of exposures or outcomes, without any external influence being applied. Longitudinal studies are often used to examine the association between participants’ exposures or treatments and outcomes over multiple follow-up periods (Liu et al., 2010; Nakai & Ke, 2009). Summarily, longitudinal studies offer diverse and comprehensive insights into the aging process, including the underpinning mechanism behind heterogeneity of later life, and the complex interplay of various psychological, cognitive, biological, and contextual factors influencing aging across the life course.

Several longitudinal studies exist in SSA, including, but not limited to, the World Health Organization Study on global AGEing and adult health (SAGE), Longitudinal Study of an INDEPTH Community, Indianapolis–Ibadan Dementia Study and Ibadan Study on Aging. These longitudinal studies are funded by global funding agencies such as the National Institute on Health and Welcome Trust Fund. However, the African government has provided initiatives funding longitudinal studies, such as the Malawi Longitudinal Study of Families and Health, the African Centre Demographic Study, the Nigerian General Household Survey Panel, and the Nakuru Eye Disease Cohort Study, to mention a few.

The use of longitudinal studies on aging in SSA has birthed several innovations across the region including development of National Policy on Ageing in more than 11 countries in the region (Saka et al., 2019), the establishment for geriatric services in countries like Nigeria (Akoria, 2016; Dotchin et al., 2013), and training of professionals in the field of aging care in the SSA (Dotchin et al., 2013). Prominent longitudinal studies on aging in SSA have highlighted the intersectionality of comorbidities associated with older adults, and access to care services in the region (Newman, 2010), for example, the Health and Aging in Africa: A Longitudinal Study of an INDEPTH Community in South Africa (HAALSI), an interdisciplinary longitudinal study provided a holistic description of the social, economic, and biological risk factors to chronic health conditions over time among older adults in South Africa (Gómez-Olivé et al., 2018). Similarly, one of the subregional longitudinal studies, Ibadan Study on Aging, which offered a new lens into the sociocultural perspectives of successful aging among Yoruba ethnic group in Nigeria (Gureje et al., 2014). Thus, creating culturally adaptable tool for assessing social support, social networks, healthy lifestyles, activities of daily living, self-reported health status, suicidality, coping, poverty, social engagement, and loneliness among older adults in South-Western region of Nigeria (Gureje et al., 2014). Although the impact of these longitudinal studies on aging is felt across regions in the SSA through policy formulations, service provision, and infrastructural development, yet these studies are marked with gaps, which includes neglecting critical issues like genetic, and genomic biomarkers of aging, mobility issues in older adults, living arrangement, environmental impact of aging, intersectionality of traditional, and religious. Despite the existence of these longitudinal studies data across the African region, it is unknown how these longitudinal studies data are being utilized. Yet there are no rigorous reviews in the region on how these longitudinal studies and data on aging have been utilized as an instrument for change in service provision for older adults, opportunities for cross-national comparison, or even promising lens for developing a hypothesis on the contribution of time, place, and societies in the trajectories of quality of life, health, and well-being of older adults in Africa.

Reviewing longitudinal studies on aging has been conducted by researchers in the United States. For instance, Stanziano et al. (2010) reviewed 51 studies from the National Institute on Aging’s Longitudinal Studies Database. They found that cognitive function, health and physical performance, socioeconomic factors, and predictors of morbidity and mortality were the most commonly studied themes, while highlighting underrepresented topics such as healthcare costs and genetics. In a similar vein, Tappen and Ouslander (2010) provided an overview of the state-of-the-art in longitudinal studies on aging, identifying three main themes: improvements in the health status of older Americans, a shift toward studying preclinical stages of diseases and their underlying mechanisms, and advancements in longitudinal study methodologies. These reviews not only suggest potential areas for future research in aging but also propose ways to advance longitudinal study methodologies and initiatives. Furthermore, these reviews underscore the critical role that longitudinal studies play in monitoring aging progress and preparing for the impact of an aging population in regions such as SSA with a projected increase in the aging population.

To the best of our knowledge, this is the first review of longitudinal studies on aging across the SSA region, while previous reviews have mapped aging studies in SSA, with one identifying gaps and offering recommendations (Kalu et al., 2021), and the other assessing the quality of reporting and methodology (Kalu et al., 2022). This current study explores longitudinal aging studies as against other forms of studies such as randomized control trials because longitudinal aging studies allow us to explore relative causality and temporal understanding (tracking changes), which are associated with the aging process and have a critical role to play in policy development, evaluation, and identification of risk factors associated with aging. Therefore, the overall aim of this review is to understand the breadth and use of longitudinal studies on aging in the SSA regions, identifying gaps, and making recommendations in preparation for the projected increase in the number of older adults in the SSA regions.

## Method

This is the third of a four-part series that aims to set priorities for aging research and practice in SSA. In the first paper, Kalu et al. (2021), provided an overview of the types and trends of aging research in the SSA context, whereas the second paper focused on the quality of reporting and methodology employed in aging research (Kalu et al., 2022). A detailed description of the methodology and procedure used in the original review has been published elsewhere (Kalu et al., 2021). Briefly, we followed the Grant and Booth (2009) analytical framework of Search, Appraisal, Synthesis, and Analysis (SALSA) to describe longitudinal aging studies and their use in SSA.

### Search Strategy

**Figure 1.**
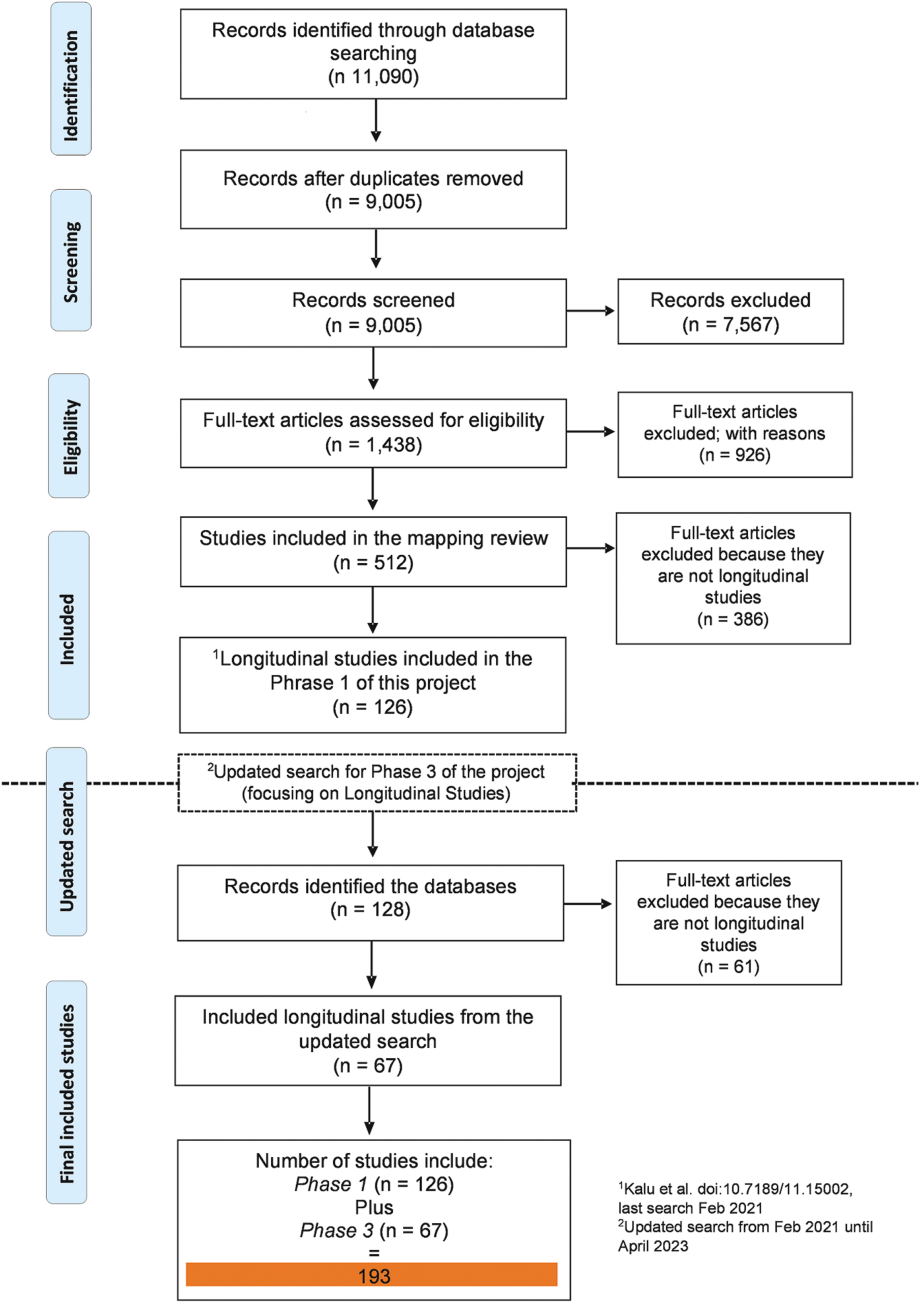
PRISMA flowchart.

The search strategy has been previously described in Kalu et al. (2021). Briefly, we conducted a thorough search of seven databases, namely CINAHL (EBSCO), PsycINFO (Ovid), EMBASE (Ovid), CENTRAL (Cochrane Library-Wiley), PEDRO, PubMed, and Web of Science using subject headings “aging” and “Sub-Saharan Africa.” In addition, we also searched the websites of longitudinal studies in Africa (if available) to identify relevant studies for inclusion. For this paper, we updated the search from December 2021 (the last date of Kalu et al.’s 2021 paper search) to April 2023, specifically focusing on longitudinal aging studies. The updated search was uploaded into Rayyan to enable study selection. Sixty-one studies met the inclusion criteria from the updated search with 126 studies from phase 1 of this project, making it a total of 193 studies (see Figure 1).

### Study Selection

Study selection was done in two processes. First, six authors independently reviewed the longitudinal studies included in previous reviews to ensure that the articles followed the description of the longitudinal study adapted for this present review. We define *longitudinal study* as a type of observational research method that involves collecting data from the same group of individuals repeatedly over an extended period of time to investigate changes in health outcomes, behaviors, attitudes, and other related variables to understand the cause and consequence of these changes based on exposure related to developmental processes and diseases progression (Liu et al., 2010; Nakai & Ke, 2009). Second, another six authors independently conducted title, abstract, and full-text screening of the output of the updated search. Authors met at each stage of title, abstract, and full-text screening, and discrepancies were discussed and resolved in a research team meeting. We included studies if: (1) the objective is focused on aging research in SSA, (2) utilized longitudinal study data that either focused on aging or a subset of longitudinal study data with older adults aged 55 years and more, (3) the name of the longitudinal study data utilized was stated, for instance WHO-SAGE, and (4) used one or more waves of the longitudinal data. Studies were excluded if (1) used data from a small number of people and followed over a period of time (e.g., randomized controlled trial with two or more follow-ups) and (2) used data from patient medical records.

### Data Extraction and Synthesis

To minimize error, we also piloted data extraction from a previous study (Kalu et al., 2021). Initially, we adapted a data extraction tool from a previous study to include variables related to longitudinal analysis, such as the type of longitudinal data, setting, study waves, and analytical approach. We piloted the modified tool with five authors, who extracted data from five studies, and then discussed the process in a research meeting to identify areas for improvement. Based on their recommendations, we refined the tool through several iterations and created two separate data extraction sheets. The first sheet focused on the primary longitudinal study, collecting information such as the study name, country, setting (rural, urban, or both), funding agencies, number of waves, start and end dates, sample size, data collection method, loss to follow-up, and study website. The second sheet captured data from articles that utilized the longitudinal study, including author names, study objectives, publication year, waves used, sample size, demographic information (e.g., age, sex, income, education, and occupation), population characteristics (e.g., older adults with HIV or heart disease), and study data analysis method—cross-sectional (using only one wave for analysis) or longitudinal (uses two or more waves for analysis).

To synthesize our findings, we employed two distinct approaches. First, we utilized frequency count, percentages, and ranges to present the demographic information, including the total number of studies and the countries in which they were conducted. Second, we analyzed the subject areas covered by researchers in their longitudinal studies and publications. To achieve this, two independent reviewers examined each study’s objectives, population, and findings, identifying the subject area in which the article was published. Both reviewers met and discussed, and a third reviewer resolved disagreements during a larger research meeting.

## Results

We identified 193 studies using 24 longitudinal studies conducted at 28 unique sites (see Supplementary Table 1). Most of the studies used WHO-SAGE, collected in South Africa and Ghana (*n* = 59, 30.5%), and HAALSI (*n* = 51, 26.4%). Four studies (Ralston et al., 2015; Schatz et al., 2018; Stringhini et al., 2018; Wilunda et al., 2015) utilized data from more than one Longitudinal study. Of the 28 longitudinal study sites, seven were conducted both in South Africa (Africa Centre Demographic Information System [ACDIS] Longitudinal Study, Cape Area Panel Study [CAPS], South African leg of the international Prospective Urban and Rural Epidemiology Study [PURE-SA-NWP], HAALSI-South Africa, Health Demographic Surveillance System [HDSS]-Agincourt, SAGE Well-being of Older peoples Study [SAGE-WOPS]-South Africa, and WHO-SAGE-South Africa); and Kenya (Demographic Surveillance System [DSS]-Kenya, HDSS-Nairobi, International Centre for AIDS Care and Treatment Program [ICAP], Millennium Villages Project [MVP], Nakuru Eye Disease Cohort Study, Survey on Social Health and Over-all Well-being of Older People, and Urbanization Poverty and Health Dynamics [UPHD]). Supplementary Table 1 shows the longitudinal studies and their characteristics, followed by the complete reference list of all the included articles. Of 193 studies, 150 used cross-sectional analysis, 42 used longitudinal analysis, and only 1 used both approaches in the same paper.

### WHO-SAGE Longitudinal Studies

We identified 59 studies that leveraged the WHO-SAGE data set, including data from baseline and waves 1 to 3. The primary goal of the WHO-SAGE study is to compile comprehensive longitudinal data on the health, well-being, and aging processes of adults from select countries, including China, Ghana, India, Mexico, Russia, and South Africa. Thirty-one studies used data from South Africa, 26 from Ghana, and five used data from South Africa, Ghana, and other non-African data (Gildner et al., 2014; Kämpfen et al., 2020; Kunna et al., 2017; McKinnon et al., 2013; Moreno-Agostino et al., 2020). Sample sizes analyzed in the included studies ranged from 23 (Charlton et al., 2020) to 10,522 (McKinnon et al., 2013). In terms of analytic approach, 52 studies adopted a cross-sectional approach, and 7 studies utilized a longitudinal approach.

The primary focus of the studies leveraging WHO-SAGE data set answered research questions relating to eight subject areas: cancer (*n* = 1), cardiometabolic disease, including stroke, diabetes, and hypertension (*n* = 9), healthcare utilization (*n* = 8), HIV (*n* = 7), mental health (*n* = 3), sleep duration and insomnia (*n* = 3), social determinants of health (*n* = 27), and vision problems (*n* = 4).

### SAGE Well-Being of Older Peoples Study

Nine studies analyzed data from the SAGE-WOPS, a three-wave longitudinal study conducted between 2010 and 2016 focused on the health, well-being, and functional status of older adults infected or affected by HIV/AIDS in Uganda and South Africa. Included studies focused on health utilization among older adults living with HIV/AIDS (Kuteesa et al., 2014; Mugisha et al., 2017), prevalence of chronic conditions (Mugisha et al., 2016), survival, stigma, and social engagement (Kuteesa et al., 2014; Mugisha et al., 2018), health status, well-being, sexual behavior (Negin et al., 2016; Nyirenda et al., 2013; Rishworth et al., 2020), and care provision and receipt among older adults infected or affected with HIV/AIDS (Mugisha et al., 2015). Of the nine studies, only one (Negin et al., 2016) employed a longitudinal analytical approach, whereas the remaining nine were cross-sectional. The sample size of the included studies ranged from 101 (Negin et al., 2016) to 932 (Nyirenda et al., 2013).

### Health and Aging in Africa: A Longitudinal Study of an INDEPTH Community

We identified 51 studies that used the HAALSI data, which include baseline, wave 1, and wave 2 data sets. The overarching aim of the HAALSI longitudinal study is to investigate the social, economic, and biological determinants of health in the older adults’ population of rural South Africa. The sample sizes from the included studies ranged from 765 (Wade et al., 2021) to 6,281 (Gaziano et al., 2017) participants. Forty-three studies employed cross-sectional analytical approach and eight utilized longitudinal analytical approach.

The studies cut across eight subject areas: mental health (*n* = 15), social determinants of health (*n* = 8), HIV (*n* = 6), cardiometabolic diseases (e.g., hypertension and diabetes, *n* = 4), sleep duration and insomnia (*n* = 3), frailty (*n* = 2), formal and informal care provision (*n* = 1), and cancer (*n* = 1).

### Ibadan Study on Aging

Twenty studies utilized data from the Ibadan Longitudinal Study (ISA). This prospective longitudinal study aims at investigating the health and well-being of older persons living in households spread across the Yoruba-speaking communities in South-Western Nigeria that have collected data in four waves between 2003 and 2009. The sample size of the included studies ranged from 201 (Ojagbemi et al., 2018) to 2,152 (Oladeji et al., 2011) participants. The analytical approach for 13 studies was cross-sectional and the remaining seven studies utilized a longitudinal approach.

The studies cut across several subject areas including mental health (e.g., depressive disorders, *n* = 4; suicidal behaviors, *n* = 1), physical health (e.g., disability, *n* = 2; falls, *n* = 1; hypertension, *n* = 1; dizziness, *n* = 1), dementia (*n* = 4), social determinants of health (*n* = 3), insomnia (*n* = 2), gait speed and cognition (*n* = 1), and hearing health (*n* = 1).

### Indianapolis–Ibadan Dementia Project

The study was a longitudinal prospective population-based comparative epidemiological study of the prevalence and incidence rates and risk factors for Alzheimer’s disease and other age-associated dementias. It was conducted among older Black adults (age >65 years) in Indianapolis (USA) and Ibadan (Nigeria) between 1992 and 1998. The total sample size at baseline was 6,770 with 2,459 community-dwelling Yoruba residents of Ibadan. After the baseline, it was followed by two waves of data collection at 2 and 5 years.

Nine studies were identified that employed data from the Indianapolis–Ibadan Dementia Study data set to investigate incidence (*n* = 3) and risk factor (*n* = 6) associated with dementia. Six studies used cross-sectional analytical approach and the remaining three studies used longitudinal analytical approach. Sample size from the included studies ranges from 108 (Baiyewu et al., 2012) to 3,275 (Gao et al., 2016).

### Health and Demographic Surveillance System

We identified 19 studies that used the HDSS: A Longitudinal Study collected from rural and urban communities in several sites in Asia and Africa including South Africa, Kenya, Tanzania, and Ethiopia. HDSS examined demographic, well-being, health information, and other information that characterizes the living conditions of older people in those communities of the studies and was collected at three waves—baseline and waves 1 and 2. The sample sizes of the included studies ranged from 1,026 (Chepngeno-Langat et al., 2019) to 13,000 (Mtowa et al., 2017) participants. All the studies employed a cross-sectional analytical approach, except four studies that used a longitudinal analytical approach (Fantahun et al., 2009; Kyobutungi et al., 2010; Mwanyangala et al., 2010; Schatz et al., 2015).

The studies cut across some subject areas: quality of life and well-being of older people (e.g., Mwanyangala et al., 2010; Wilunda et al., 2015), sociodemographic inequalities in HIV testing and HIV prevalence in older people (e.g., Mtowa et al., 2017), living conditions and household arrangement (e.g., Chepngeno-Langat et al., 2019; Kyobutungi et al., 2010; Schatz et al., 2015, 2018; Wilunda et al., 2015), and cardiovascular disease (CVD) management (e.g., Jardim et al., 2017, 2018).

### Other Longitudinal Studies

We found 25 studies used various longitudinal studies ranging from 1 to 4 studies per longitudinal studies. Four and three studies utilized DSS-Tanzania, Kenya) and MVP, respectively. Two studies each utilized ACDIS Longitudinal Study, International Center for AIDS Care and Treatment Program, Columbia University (ICAP), Malawi Longitudinal Study of Families and Health; Survey on Social, Health and Over-all Well-being of Older People, and Well-being of Older Peoples’ Study. One study each utilized Agence Nationale de la Statistique et de la Démographie, CAPS, and CLARIFY, South African leg of the international Prospective Urban and Rural Epidemiology Study, Kagera Health and Development Survey (KHDS), Nakuru Eye Disease Cohort Study, Nigeria General Household Survey Panel (Nigeria GHS-Panel), the Gambian Bone and Muscle Ageing Study (GamBAS), Ugandan Non-Communicable Diseases and Aging Cohort Study, and UPHD. Six studies used the longitudinal analytic approach, while the remaining 19 studies used the cross-sectional analytical approach.

The studies covered many subject areas including HIV and AIDS (*n* = 6), aging and health (*n* = 3), social determinant of health (*n* = 1), caregiving (*n* = 4), CVD (*n* = 1), frailty (*n* = 1), mortality (*n* = 2), hypertension (*n* = 1), and noncommunicable disease (*n* = 1). Details of each study are shown in Supplementary Table 1, while Table 1 provided summaries of the Longitudinal Aging Studies included in this review.

**Table 1.** Description of Included Longitudinal Studies of Aging in Sub-Saharan Africa (*n* = 24)

Name of Longitudinal Study	Country	Website or main reference article	No. of studies that utilized the data set
Africa Centre Demographic Information System (ACDIS) Longitudinal Study	South Africa	https://ghdx.healthdata.org	2
Agence Nationale de la Statistique et de la Démographie	Senegal	Yakam et al., 2022	1
Cape Area Panel Study (CAPS)	South Africa	University of Cape Town and University of Michigan, 2012	1
CLARIFY^a^	45 countries including South Africa	Steg, 2009	1
Demographic surveillance system (DSS)-(Tanzania)	Tanzania	Geubbels et al., 2015	3
The Kilifi Health and Demographic Surveillance System (KHDSS)	Kenya	Scott et al., 2012	1
Epidemiology of Demetia in Central Africa (EPIDEMICA)	Central African Republic and Republic of Congo	Guerchet et al., 2014	3
South African leg of the international Prospective Urban and Rural Epidemiology Study (PURE-SA-NWP)	South Africa and Zimbabwe	https://www2.phri.ca/pure/	1
HAALSI (Health and Aging in Africa: A Longitudinal Study of an INDEPTH Community in South Africa) Study	South Africa	http://www.indepth-network.org/projects/haalsi	51
Health Demographic Surveillance System (HDSS)	South Africa, Kenya, Tanzania, and Ethiopia	http://www.indepth-network.org/	32
Ibadan Study of Aging (ISA)	Nigeria	Gureje et al., 2015	20
International Center for AIDS Care and Treatment Program, Columbia University (ICAP)	Kenya	https://icap.columbia.edu/	2
Indianapolis–Ibadan Dementia Project (IIDP)	Nigeria and Indianapolis	https://iidpportal.medicine.iu.edu/	9
Kagera Health and Development Survey (KHDS)	Tanzania	https://microdata.worldbank.org/index.php/catalog/359 https://microdata.worldbank.org/index.php/catalog/79 https://microdata.worldbank.org/index.php/catalog/2251	1
Malawi Longitudinal Study of Families and Health	Malawi	Kohler et al., 2020	2
Millennium Villages Project (MVP)	Kenya	Sachs, 2018 https://www.un.org/esa/coordination/Alliance/ Earth%20Institute%20-%20The%20Millennium%20Villages%20Project.htm	3
Nakuru Eye Disease Cohort Study	Kenya	Bastawrous et al., 2014	1
Nigeria General Household Survey Panel (Nigeria GHS-Panel).	Nigeria	https://nigeria.opendataforafrica.org/	2
Survey on Social, Health and Over-all Well-Being of Older People	Kenya	https://microdataportal.aphrc.org/index.php/catalog/48	2
The Gambian Bone and Muscle Ageing Study (GamBAS)	Gambia	https://thesamson.org/the-gambian-bone-ageing-study/	4
Ugandan Non-Communicable Diseases and Aging Cohort Study	Uganda	Siedner et al., 2021 https://clinicaltrials.gov/ct2/show/NCT02445079	1
Urbanization Poverty and Health Dynamics (UPHD)	Kenya	Soldo et al., 1997	1
SAGE Well-Being of Older Peoples Study (SAGE-WOPS)-Uganda and South Africa	Uganda and South Africa	https://www.who.int/data/data-collection-tools/study-on-global-ageing-and-adult-health/sage-wops-hiv-waves	9
WHO Study on Global Ageing and Adult Health (WHO-SAGE)	Several countries including South Africa and Ghana	https://apps.who.int/healthinfo/systems/surveydata/index.php/catalog/sage/about	*✝*59

*Notes*: ^✝^Some studies used longitudinal studies/data from more than one country.

^a^CLARIFY includes samples across 45 countries including South Africa.

## Discussion

The importance of longitudinal aging studies cannot be overemphasized, as they provide the opportunity to understand the complex mechanism associated with the aging process illuminating the biopsychosocial influences on several health and social outcomes across the life course. Our review highlighted that longitudinal aging studies in Africa focused primarily on understanding the biological/medical process or conditions associated with aging, highlighting the gaps in a comprehensive understanding of social and contextual conditions, such as environment, financial, and personal conditions influencing the aging process in SSA. In SSA, issues affecting aging are rooted in regrettable and implicit social and contextual conditions. For instance, social warfare for older adults’ care is almost nonexistent in some nations because of several reasons, including but not limited to inadequate government resources and funding, inadequate policy frameworks, and cultural beliefs and attitudes towards older adults (Aboderin & Beard, 2015; Aboderin & Ferreira, 2008; Adamek et al., 2021; National Research Council, 2006; Saka et al., 2019). A 2022 study reported that 72 gerontological scholars from 17 countries stated that the lack of social services targeted at older adults could result from competing priorities in addressing other societal needs for other populations (Adamek et al., 2021). Our finding, alongside previous evidence, highlighted the need for aging research in SSA to refocus on social- and contextual-related areas, identifying the economic and social inequalities that may contribute to lack of resources and support for the older adult’s population.

A synthesis of the articles showed a skewed focus of data sets on urban rather than rural areas. For example, out of all the longitudinal data sets, only three integrated rural and urban-dwelling older adults in their study. One of them (HDSS: A Longitudinal Study) focused on rural- and urban-dwelling older adults. The other two (Indianapolis–Ibadan Dementia Study data set and HAALSI) focused only on community-dwelling older adults. This finding resonates with a previous scoping review where it was reported that studies in Africa tend to focus more on the urban than the rural settings, even though most of the older adults reside in rural communities (Kalu et al., 2021). This tends to question the applicability of the findings of aging research in the rural setting because most aging research is conducted in urban settings. More so, focusing aging research on the urban communities in Africa creates room for exclusion and underrepresentation of rural-dwelling older adults in research and, consequently, in programs and policies. Therefore, it is crucial that future research and longitudinal data set specifically focus on older adults in rural communities, as this could facilitate knowledge of the aging context of this population and specific ways to improve their general aging experiences.

Furthermore, gender disparity in research participation is another finding that raises concern on how that affects the aging experiences of older male and female in Africa. In this study and elsewhere, older Black men are less represented in research (Ojembe & Kalu, 2018). For example, one of the included studies in this review using the Kilifi Health and Demographic Surveillance System (KHDSS), included 6.8% male in 2001 and 9.6% male in 2011 as against female respondents representing 11.1% in 2001 and 14.6% in 2011 (Scott et al., 2012). This trend is unsettling and raises questions on factors encumbering the participation of older Black men in research and its implication in aging experiences of older men in Africa. In essence, the underrepresentation of older Black men in research stymies the explication of ways to improve their quality of life. Although recent research is starting to pay attention to increasing the representation of Black men in research (Ebimgbo et al., 2021), there is a need for future research to adopt a more maximally varied recruitment approach to facilitate the understanding of strategies to address the differing needs of both men and women.

An interesting finding from our study is the commendable positions of South Africa and Kenya in taking the lead in longitudinal aging studies in SSA. Of the 23 longitudinal studies, 8 were conducted in South Africa and 7 in Kenya. Longitudinal studies such as the WHO-SAGE, SAGE-WOPS, CAPS, PURE, HAALSI, and HDSS were conducted in SSA, whereas MVP, ICAP, HDSS, UPHOLD, and the Nakuru Eye Disease Cohort Study were conducted in Kenya. This number of longitudinal studies in these countries might be related to some factors. For example, South Africa has one of highest proportion of older population in SSA and falls just below Mauritius and Seychelles (Kalu et al., 2021). South Africa and Kenya also appear to have more availability of research funding from international sponsors such as WHO, United Nations, U.S. National Institute on Aging, and the Wellcome Trust, UK. This is in addition to the apparent extensive research collaborations by South African and Kenyan research institutions, such as the University of KwaZulu-Natal, the University of Cape Town, and the African Population and Health Research Center Nairobi, and international research organization, such as University of Michigan and Harvard University. Although collaborations for longitudinal studies with well-established centers in Global North do exist in countries with the highest projected increase in the number of older adults in SSA, like Nigeria, these collaborations often need to be expanded in scope. For example, in Nigeria, the Indianapolis–Ibadan Dementia Study collaboration is restricted to the southwest region and does not include older adults from the other five regions, representing a substantial portion of the aging population. To improve the comprehensiveness of data on aging populations and inform national policies on aging in Nigeria, it would be valuable to expand these collaborations to cover more regions of the country. Similar efforts can also be implemented in other SSA countries, including Uganda, Mauritius, and Seychelles, which also have high numbers of older adults.

Longitudinal data are used to understand the temporal relationship between exposure and outcome. However, approximately 77% of the studies in our review conducted only a cross-sectional analysis of either the baseline data or one of the waves of the longitudinal data set. This finding is not entirely surprising as previous studies have reported inaccurate analysis of longitudinal data (Caruana et al., 2015). A cross-sectional analysis approach inherently limits the usefulness of the longitudinal data, especially when there are multiple data points available. Using inappropriate analysis or underanalyzing longitudinal data is often detrimental to the evidence these data can provide on the aging population and health of the population. We argue that the complexity of longitudinal data analysis, which includes structural modeling or trajectory pattern analysis that often require a sound knowledge of specific statistical software and skill, may be one reason why the analysis of longitudinal aging data in Africa is limited (Anstey & Hofer, 2004). Additionally, issues such as missing data, unequal distribution of sample sizes across waves, irregular time data collection, and limited resources could contribute to the lack of such analysis in SSA (Garcia & Marder, 2017). Resources are a crucial factor and may be strong limiting factors in the utilization of longitudinal data in SSA. Moreso, the INDEPTH network identified the low level of data analysis utilized by researchers in SSA and attributed it to lack of capacity (Mbacké & Phillips, 2008). Although researchers from different parts of the world utilize these longitudinal study data sets, a significant number were from SSA. To promote the utilization of longitudinal data for addressing longitudinal research questions in SSA, we suggest that training institutions in the region, especially at the graduate level, could design specialized courses on longitudinal data analysis to enhance skills and knowledge. Additionally, providing continuing education and training opportunities, backed by funding, can also be effective in improving capacity for longitudinal data analysis.

Although our review had a comprehensive search strategy and utilized multiple coders at different stages of study selection, data extraction, and analysis, there are some limitations to consider. Non-English language publications may have been missed, and some longitudinal studies, such as the ISA, may have been overlooked due to a lack of a website attached to the data set. We attempted to contact the principal investigators of these studies without functional websites to confirm that we did not miss any articles but received no response. Also, we used a search strategy from our previous reviews, including the names of some longitudinal study data sets in the search term, could have broadened our search results. However, our study provides a useful summary of aging longitudinal studies in SSA, which can serve as a foundation for future research.

### Recommendations

To improve longitudinal aging research in SSA, we recommend the following:

(a) International collaborations with SSA countries with a projected to have higher increase in the older adult population, such as Nigeria, Mauritius, and Seychelles. One of the criteria for this collaboration should include the availability of data for other use by other researchers rather than the principal investigators.
(b) Extending longitudinal studies to include a focus on social, financial, and contextual factors influencing the aging process; and in the rural community incorporating maximum variation sampling for age, gender, and another socioeconomic status
(c) National and international funding calls to encourage use of longitudinal data sets and analysis, such as trajectory modeling, to identify aging profiles and transitional patterns between premorbid and morbid states in the SSA population. This advanced approach will provide context-dependent intervention to tackle premorbid states, such as preclinical disability, to advance health promotion, reducing aging with disability among the SSA population.
(d) Continuous cross-country training and internships between African countries with well-established research-focused aging centers, such as South Africa, Kenya, Nigeria, and Ghana, to build capacity for the aging research.

## Conclusion

We included 193 studies that used data from 24 longitudinal studies conducted at 28 unique sites across SSA. The most commonly used longitudinal data sets were WHO-SAGE and HAALSI; however, most studies analyzed the data cross-sectionally rather than longitudinally. Most longitudinal studies focused on aging-related biological/physical or medical processes. Few studies focused on social processes and limited attention to other contextual factors, such as environmental and financial factors influencing the aging process across the life course. Implementing the above recommendation is promising to build capacity for aging research and practice in preparation for the projected aging population in SSA. The last paper of this series will focus on identifying priorities for aging research in SSA through an e-Delphi process across international researchers, practitioners, older adults, and family caregivers residing in SSA countries.

## Supplementary Material

igae002_suppl_Supplementary_Table_S1
