# Supplementary material for: Longitudinal Studies of Aging in Sub-Saharan Africa: Review, Limitations, and Recommendations in Preparation of Projected Aging Population
Source: Innov Aging. 2024 Jan 23;8(4):igae002. doi: 10.1093/geroni/igae002 (PMC11020233; doi:10.1093/geroni/igae002)
Supplement: igae002_suppl_Supplementary_Table_S1 [file igae002_suppl_supplementary_table_s1.docx]

**Supplementary Table 1.** Characteristics of included longitudinal studies (n=193).

| **Study** | **Country** | **Study Objective** | **Name of Longitudinal Study** | **Sample Size** | **Mean Age (SD) or Age Limit** | **Analytic Approach** |  |
| --- | --- | --- | --- | --- | --- | --- | --- |
| Ackuaku-Dogbe et al. (2015) | Ghana | To describes cataract surgical uptake among older adults in Ghana. | WHO SAGE | 5571 | 50+ | Cross-sectional |  |
| Adams et al. (2020) | South Africa | To validate the CES-D among an aging Shangaan-speaking and predominantly Black African sample in rural South Africa, with a special emphasis on gender differences | HAALSI | 5059 | 40+ | Cross-sectional |  |
| Adhvaryu & Beegle (2012) | Tanzania | To provide empirical evidence of the impacts of adult deaths on older adults in Kagera, northwest Tanzania. | KHDS | 613 | 52.5 (10.1) | Longitudinal |  |
| Aheto et al. (2020) | Ghana | To examine socio-demographic and environmental determinants associated with asthma in older adults in Ghana. | WHO SAGE | 4621 | 50+ | Cross-sectional |  |
| Akuamoah-Boateng (2013a) | Ghana | To analyze vision health and its determinants among the older adult population in a district in one of the poorest regions in Ghana – the Kassena-Nankana district. | WHO SAGE | 4294 | 50+ | Longitudinal |  |
| Amegbor et al. (2018) | Ghana | To determine the predictors of basic self-care and intermediate self-care functional disabilities among older adults in Ghana. | WHO SAGE | 4107 | 50+ | Cross-sectional |  |
| Amegbor et al. (2022) | Ghana | To examine how chronic health conditions, functional limitations, socioeconomic status, and place of residence predict overnight hospital admission as well as the frequency of admission among older adults in Ghana | WHO SAGE | 5573 | 50+ | Cross-sectional |  |
| Amegbor et al. (2020b) | Ghana | To investigate the effect of cognitive social capital (trust and sense of safety) and structural social capital (social participation or engagement in social activities) on depression among older adults in Ghana. | WHO SAGE | 4123 | 50+ | Cross-sectional |  |
| Amegbor et al. (2021) | Ghana | To examine the association between social frailty and depression among older adults in Ghana over time | WHO SAGE | 4732 | 50+ | Cross-sectional |  |
| Annin et al. (2014) | Ghana | To examine the association between the degree of pain and socioeconomic status among older male and female Ghanaians. | WHO SAGE | 5108 | 50+ | Cross-sectional |  |
| Ardington et al. (2010) | South Africa | To demonstrate the role played by aging parents in caring for grandchildren who lose parents due to illnesses such as AIDS. | CAPS | 696 | 60 (NR) | Cross-sectional |  |
| Asiimwe et al. (2020) | South Africa | To describe the population distribution of cognitive outcomes in a community of older PLWH residing in South Africa. | HAALSI | 4560 | 40+ | Cross-sectional |  |
| Awoke et al. (2017) | Ghana | To identify factors associated with public and private healthcare utilization among older adults aged ≥50 years in Ghana, and compare perceived differences in health system responsiveness between the private and public sectors. | WHO SAGE | 2517 | NR | Cross-sectional |  |
| Ayernor (2012) | Ghana | To outline the chronic non-communicable disease burden of older adults and predict the odds of living with a chronic non-communicable disease in Ghana. | WHO SAGE | 507 | NR | Cross-sectional |  |
| Baiyewu et al. (2007) | Nigeria, USA | The comparative prevalence of depression in the two communities is reported, as well as the association of the depressive symptoms with age, gender and cognitive performance. | IIDP | 1058 | 78.8 (6.2) | Cross-sectional |  |
| Baiyewu et al. (2012) | Nigeria | To assess the level of neuropsychiatric symptoms in community-dwelling individuals with dementia, cognitive impairment no dementia and normal cognition. | IIDP | 108 | 80 (NR) | Cross-sectional |  |
| Barker et al. (2021) | South Africa | The study aims were firstly to develop a cumulative deficits frailty index using a large population-based cohort from South Africa, and secondly to test whether such a frailty index is associated with increased mortality and worsened subjective wellbeing. | HAALSI | 3989 | 61.3 (NR) | Cross-sectional |  |
| Balogun et al. (2018) | Nigeria | To examine the determinants of bed net use among older Nigerian adults. | Nigeria General Household Survey-Panel | 3439 | 61.4 (10.2) | Cross-sectional |  |
| Bastawrous et al. (2016) | Kenya | To describe the cumulative 6-year incidence of visual impairment (VI) and blindness in an adult Kenyan population. | Nakuru Eye Disease Cohort Study | 4414 | 62.5 (9.3) | Longitudinal |  |
| Bekibele & Gureje (2010) | Nigeria | To examine the prevalence and factors associated with falls among a population of elderly persons in Nigeria | ISA | 2096 | NR | Cross-sectional |  |
| Bennett et al. (2016) | Kenya | To examine gender differentials in survival amongst older people (50+ years) in the Nairobi slums. | NUHDSS | 2972 | NR | Cross-sectional |  |
| Biritwum et al. (2013) | Ghana | To provide a framework that is capable of transforming and improving the lives of older persons in the Ghanaian society. | WHO SAGE | 5000 | NR | Longitudinal |  |
| Boateng et al. (2017) | Ghana | To examine the relationship between obesity and the risk of chronic diseases, cognitive impairment, and functional disability among the elderly in Ghana. | WHO SAGE | 2091 | NR | Longitudinal |  |
| Calys-Tagoe et al. (2014) | Ghana | To examine the lived experiences of older people in Ghana | WHO SAGE | 4724 | 64.2 (10.7) | Longitudinal |  |
| Calys-Tagoe et al. (2020) | Ghana | To report the prevalence of hypertension, its awareness and treatment effectiveness among older adults (aged 50 years and above) in Ghana. | WHO SAGE | 3575 | 65.1 (10.7) | Cross-sectional |  |
| Chang et al. (2019a) | South Africa | To examine how multimorbidity might affect progression along the continuum of care among older adults with hypertension, diabetes and human immunodeficiency virus (HIV) infection in rural South Africa. | HAALSI | 4447 | NR | Cross-sectional |  |
| Chang et al. (2019b) | South Africa | To enhance the evidence base on multimorbidity by developing a comprehensive portrait of the epidemiology of multimorbidity in a rural South African community. | HAALSI | 3889 | NR | Cross-sectional |  |
| Charlton et al. (2020) | South Africa | To evaluate the impact of collecting three consecutive repeated 24 h samples for calculating the correction factor for urinary Na excretion and comparing this with the effect of averaging the results for each person. | WHO SAGE | 23 | 59.7 (15.6) | Longitudinal |  |
| Chepngeno-Langat (2013) | Kenya | To examine HIV risk perception and correlates of perceived risk among older people aged 50 years and older living in Nairobi slums. | NUHDSS | 2053 | 59.1 (NR) | Cross-sectional |  |
| Chepngeno-Langat (2014) | Kenya | To examine transition in and out of the care-giving role and whether the likelihood of becoming a caregiver is influenced by socio-demographic and socioeconomic factors; and also to understand the relationship between care-giving transitions and the health and financial status of the caregiver. | UPHD | 1485 | 58.5 (8.4) | Longitudinal |  |
| Chepngeno-Langat et al. (2011) | Kenya | To investigate the association between caregiving and poor health among older carers to people living with AIDS, and examine potential within-gender differences in reporting poor health. | DSS-Kenya | 1429 | 59.6 (NR) | Cross-sectional |  |
| Chepngeno-Langat et al. (2012) | Kenya | To explore the way that social networks and personal experiences affect perceived HIV-related concerns among people aged 50 years or older living in a low resource neighborhood with high HIV prevalence in Nairobi, Kenya. | Survey on Social, Health and Overall Wellbeing of Older People study | 2061 | 59.1 (NR) | Longitudinal |  |
| Chepngeno-Langat et al. (2019) | Kenya | To examine how recipients of the Kenyan Older Persons Cash Transfer Program (OPCTP) living in two slum communities in Nairobi reallocate their social pension by examining the characteristics of older people who are more likely to share their cash and identifying secondary beneficiaries. | NUHDSS | 1026 | NR | Cross-sectional |  |
| Clark et al. (2014) | Nigeria | To compare the effect of obesity and related risk factors on 10-year mortality in two cohorts of older adults of African descent; one from the United States and one from Nigeria. | IIDP | 2466 | 77.1 (5.3) | Longitudinal |  |
| Dei & Sebastian (2018) | Ghana | To ascertain whether horizontal and vertical equity requirements were being met in the healthcare utilization among older adults aged 50 years and above. | WHO SAGE | 4304 | NR | Cross-sectional |  |
| Eduardo et al. (2014) | Kenya, Tanzania, Rwanda and Mozambique | To estimate the proportion of newly enrolled and active adult patients receiving HIV care and initiating ART who were ≥50, and to compare baseline characteristics and outcomes between PLWH ≥50 and PLWH <50 years in sub-Saharan Africa. | ICAP | 38337 | NR | Longitudinal |  |
| Fantahun et al. (2009) | Ethiopia | To examine trends in survival to old age and identify the factors associated with longevity among the elderly (age 65 years). | Demographic Surveillance System-Ethiopia | 2231 | NR | Longitudinal |  |
| Farrell et al. (2020) | South Africa | To evaluates education and literacy as primary drivers of gender equality in cognitive performance among middle-aged and older adults in rural South Africa. | HAALSI | 2144 | 59.8 (NR) | Cross-sectional |  |
| Ferrari et al. (2015) | 45 Countries | To ascertain the current prevalence and control of key cardiovascular risk factors in outpatients with stable coronary artery disease (CAD) worldwide. | CLARIFY | 32954 | 64.2 (10.5) | Cross-sectional |  |
| Ferro et al. (2021) | South Africa | To characterize the spectrum of abnormalities suggesting end-organ damage on ECG and transthoracic echocardiograms (TTE) among older adults with cardiovascular diseases in rural South Africa | HAALSI | 5059 | 54.7 (NR) | Cross-sectional |  |
| Gao et al. (2015) | Nigeria, USA | To examine the relationship between cholesterol and other lipids APOE genotype, and risk of Alzheimer disease (AD) | IIDP | 3275 | Wave 1 = 77.7 (5.9); wave 2 = 77.2 (5.5) | Longitudinal |  |
| Gatimu et al. (2016) | Ghana | To investigate the prevalence and risk factors for diabetes in persons aged 50 years and older in Ghana. | WHO SAGE | 5565 | NR | Cross-sectional |  |
| Gaziano et al. (2017) | South Africa | To investigate the impact of life expectancy on the cardiometabolic disease mortality and morbidity among middle-aged and older adults. | HAALSI | 6281 | 61.7 (13.1) | Cross-sectional |  |
| Geldsetzer et al. (2019) | South Africa | The study measured several health outcomes and was not powered to any specific outcome or exposure. Instead, the sample size was chosen to be able to detect effect sizes of clinical relevance for nonrare exposures (eg, a prevalence of at least 10%) | HAALSI | 5059 | 62.0 (NR) | Longitudinal |  |
| Gildner et al. (2014) | 6 Countries | The study hypothesized that short sleep durations will be positively associated with obesity; and, Higher subjective sleep quality ratings will be inversely related to obesity risk. | WHO SAGE | 28717 | NR | Cross-sectional |  |
| Gómez-Olivé et al. (2013) | South Africa | To assess the relationship between self-reported chronic health conditions and chronic disease risk factors, such as smoking and alcohol consumption, and health-care utilization in a rural South African population aged ≥50. | WHO SAGE | 425 | NR | Cross-sectional |  |
| Gómez-Olivé et al. (2014a) | South Africa | To describe the mortality trends and identify possible HIV-related trends, and to identify the social and functional risks related to increased mortality risk in this cohort. | WHO SAGE | 4085 | 66.1 (NR) | Cross-sectional |  |
| Gómez-Olivé et al. (2014b) | South Africa | To examine the prospective association of sleep problems with mortality among older adults in rural South Africa, as well as potential sex differences in this association. | DSS-Agincourt | 4044 | 66.0 (11.0) | Cross-sectional |  |
| Gómez-Olivé et al. (2018) | South Africa | To examine the association between sleep parameters, non-communicable disease, HIV status and medications in older rural south African | HAALSI | 5059 | 61.0 (NR) | Cross-sectional |  |
| Gray et al. (2016) | Tanzania | To present three-year mortality rates and to identify mortality predictors in a cohort of elderly community dwellers in rural northern Tanzania. | DSS-Tanzania | 2232 | 77.9 (NR) | Cross-sectional |  |
| Gray et al. (2017) | Tanzania | To determine the feasibility of developing a short frailty screening instrument for use in low- and middle-income countries. | DSS-Tanzania | 1198 | NR | Cross-sectional |  |
| Gureje et al. (2006a) | Nigeria | This study aimed to determine the profile of dementia in a sub-Saharan Africa country and assess its effects on role functioning and quality of life | ISA | 2152 | NR | Cross-sectional |  |
| Gureje et al. (2006b) | Nigeria | This article reports estimates of disability in a regionally representative sample of persons aged 65 and older in Nigeria | ISA | 2149 | 75.0 (9.2) | Cross-sectional |  |
| Gureje et al (2006c) | Nigeria, USA | To compare dementia incidence of African-American and Yoruba cohorts aged 70 years enrolled in 1992 and 2001. | IIDP | 2245 | 82.4 (9.3) | Cross-sectional |  |
| Gureje et al. (2007) | Nigeria | To estimate the occurrence and effect of major depressive disorder in a large and representative community sample of elderly Africans | ISA | 2152 | 51.0 (18.3) | Cross-sectional |  |
| Gureje et al. (2008a) | Nigeria | To compare the effects of depression and chronic physical conditions on disability in elderly persons. | ISA | 2152 | NR | Cross-sectional |  |
| Gureje et al. (2008b) | Nigeria | We were particularly interested in examining the relative salience of social factors, compared to economic and health factors, to different aspects of QoL of elderly persons living in a society undergoing rapid social changes | ISA | 2152 | NR | Cross-sectional |  |
| Gureje et al. (2009) | Nigeria | To provide information on the profile, comorbidity and impact of insomnia among an understudied group of elderly Africans | ISA | 2152 | NR | Cross-sectional |  |
| Gureje et al. (2011a) | Nigeria | To describe the incidence of dementia in a representative sample of elderly Yoruba Nigerians and provide information about the risk factors. | ISA | 2149 | NR | Cross-sectional |  |
| Gureje et al. (2011b) | Nigeria | To determine the incidence and risk factors for insomnia among an under-studied population of elderly persons in Sub-Saharan Africa. | ISA | 2149 | NR | Cross-sectional |  |
| Gureje et al. (2014) | Nigeria | To determine the profile and determinants of successful aging in a developing country characterized by low life expectancy and where successful agers may represent a unique group. | ISA | 2149 | NR | Cross-sectional |  |
| Hall et al. (2006) | Nigeria, USA | To examine the relationship between cholesterol and other lipids APOE genotype, and risk of Alzheimer disease (AD) | IIDP | 1075 | 71.7 (6.3) | Cross-sectional |  |
| Harling et al. (2020a) | South Africa | To analyze the degree to which care needs are met in an aging rural African population | HAALSI | 5059 | NR | Cross-sectional |  |
| Harling et al. (2020b) | South Africa | To investigate how pattern of social contact social support and cognitive health in rural south africa fit with three theories | HAALSI | 5059 | NR | Cross-sectional |  |
| Hendrie et al. (2014) | Nigeria, USA | To test whether APOE e4 is associated with cognitive decline in both Indianapolis and Ibadan sample | IIDP | 4071 | NR | Cross-sectional |  |
| Hendrie et al. (2004) | Nigeria, USA | To describe the construction of a disease model incorporating both genetic and environmental factors in the etiology of Alzheimer’s disease, using data generated from the Indianapolis-Ibadan Dementia Project | IIDP | 1381 | NR | Longitudinal |  |
| Heyns et al. (2011) | South Africa | To compare the presenting features and management of prostate cancer among different racial groups. | HAALSI | 5059 | NR | Cross-sectional |  |
| Hosegood & Timaeus (2005) | South Africa | This paper examines changes in households with older people in a northern rural area of KwaZulu Natal province, South Africa | ACDIS | 3657 | NR | Cross-sectional |  |
| Houle et al. (2019) | South Africa | Aimed to estimate baseline associations between cognitive function and cardiometabolic disease risk factors in rural South Africa. | HAALSI | 3018 | 59.0 (NR) | Cross-sectional |  |
| Humphreys et al. (2017) | South Africa | To (a) assess validity of the Oxford Cognitive Screen (OCS-Plus), a domain-specific cognitive assessment designed for low-literacy settings, especially in low- and middle-income countries (LMIC); and (b) advance theoretical contributions in cognitive neuroscience in domain-specific cognitive function and cognitive reserve, especially related to dementia. | HAALSI | 1402 | NR | Cross-sectional |  |
| Ice et al. (2010) | Kenya | To examine the impact of caregiving on health using longitudinal data. | Kenyan Grandparents Study | 611 | NR | Longitudinal |  |
| Jardim et al. (2017) | South Africa | To assess cardiovascular disease (CVD) management in a rural community in northeast South Africa | DSS-Agincourt | 5059 | 61.7 (NR) | Cross-sectional |  |
| Jardim et al. (2018) | South Africa | To characterize the cardiovascular disease (CVD) profile of individuals aged 80 and older in rural South Africa. | DSS-Agincourt | 5059 | NR | Cross-sectional |  |
| Jennings et al. (2022) | South Africa | To investigate the associations between marital experiences and depressive symptoms, by gender, and explored whether economic resources is a moderator of these associations. | HAALSI | 4176 | Women: 61.3 (NR); Men: 61.7 (NR) | Cross-sectional |  |
| Jennings et al. (2021) | South Africa | The study investigates how caregiving for grandchildren is associated with cognitive function among rural South Africans, and whether the association differs by gender. In addition, it further investigate whether measures of physical activity or social engagement mediate this association | DSS-Agincourt | 3668 | NR | Cross-sectional |  |
| Jennings et al. (2020) | South Africa | To explore how receipt of emotional support differs by gender and marital status | HAALSI | 5059 | 61.7 (NR) | Cross-sectional |  |
| Kämpfen et al. (2020) | Ghana, India and Russia | The purpose of this paper is twofold. First, we use a range of surveys on aging from the wider international family of ‘‘Health and Retirement Studies’’ to document variation in physical health as measured by handgrip strength across countries with different levels of economic development. | WHO SAGE | 99915 | NR | Cross-sectional |  |
| Kimuna & Makiwane (2007) | South Africa | The objective of this study was to examine the changing role of older people due to changes in household structure and the availability of an old age pension. | Mpumalanga Older People's Survey | 1002 | NR | Cross-sectional |  |
| Kobayashi et al. (2017) | South Africa | To investigate the relationships of self-rated childhood health and father’s occupation during childhood with later-life cognitive function score; and whether educational attainment mediated these relationships among older, rural South Africans living in a former region of Apartheid-era racial segregation. | HAALSI | 5059 | 61.7 (13.1) | Cross-sectional |  |
| Kobayashi et al. (2019a) | South Africa | We therefore aimed to estimate the independent associations between each of household wealth and consumption quintiles and four chronic disease risk behaviors (moderate-to-vigorous intensity physical activity, overweight/obese body mass index, smoking, and frequent alcohol intake) in a population-based study of older adults in rural South Africa. | HAALSI | 5059 | 40+ | Cross-sectional |  |
| Kobayashi et al. (2019b) | South Africa | To estimate the prevalence of cognitive impairment, and the sociodemographic and co-morbid predictors of cognitive function among older, rural South African adults. | HAALSI | 4778 | 75.0 (NR) | Cross-sectional |  |
| Kobayashi et al. (2019c) | South Africa | To estimate the relationship between height (a measure of early-life cumulative net nutrition) and later-life cognitive function among older rural South African adults, and whether education modified this relationship. | HAALSI | 5059 | 61.7 (13.1) | Cross-sectional |  |
| Kobayashi et al. (2020) | South Africa | To investigate associations between adverse childhood experiences and cognitive function in an older population who grew up under racial segregation during South African apartheid. | HAALSI | 1871 | 58.9 (NR) | Cross-sectional |  |
| Kobayashi et al. (2021) | South Africa | To determine the incidence of cognitive impairment and its distribution across key demographic, social, and health-related factors among older adults in rural South Africa | HAALSI | 5059 | 60.1 (12.2) | Cross-sectional |  |
| Kunna et al. (2017) | China and Ghana | This study measures and decomposes socio-economic inequality in single and multiple NCD morbidity in adults aged 50 and Over in China and Ghana | WHO SAGE | 4050 | 50+ | Cross-sectional |  |
| Kuteesa et al. (2014) | Uganda | To describe the experiences of stigma and disclosure in a cohort of HIV-positive older people in Uganda. | SAGE WOPS | 183 | 59.0 (NR) | Cross-sectional |  |
| Kuuire et al. (2021) | Ghana | The study examined gendered nuances in how health insurance coverage influenced unmet health-care need among older persons in Ghana, | WHO SAGE | 1245 | 76.0 (NR) | Cross-sectional |  |
| Kyobutungi et al. (2010) | Kenya | To describe the health and wellbeing of older people in two Nairobi Slums | DSS-Kenya | 2696 | 59.2 (9.1) | Longitudinal |  |
| Lartey et al. (2019) | Ghana | To examine recent changes in obesity prevalence and associated factors for older adults in Ghana between 2007/8 and 2015/15 | WHO SAGE | 4158 | 64.3 (10.8) | Cross-sectional |  |
| Lasisi & Gureje (2014) | Nigeria | This longitudinal cohort study determines the prevalence of dizziness and the clinical and socio-epidemiological correlates in elderly persons residing in the Yoruba-speaking areas of Nigeria. | ISA | 1299 | 77.3 (NR) | Longitudinal |  |
| Lasisi et al. (2010) | Nigeria | To determine the prevalence and correlates of tinnitus among community elderly and its impact on their quality of life | ISA | 1302 | 77.3 (NR) | Longitudinal |  |
| Lee & Schafer (2023) | South Africa | To examine how structural, compositional and functional aspect of older adults close social network are associated with HIV testing in South Africa | HAALSI | 5059 | 65.8 (NR) | Longitudinal |  |
| Lloyd-Sherlock et al. (2020) | South Africa | To investigate the effects of receiving a pension on reported food scarcity, body mass index and patterns of consumption. | HAALSI | 2701 | 60+ | Longitudinal |  |
| Macia et al. (2015) | Senega | The aim of the study was to assess the links between socio-demographic factors, economic conditions, health, social relations, and the life satisfaction of older adults in Dakar. | Agence Nationale de la Statistique et de la Démographie dating from the last census (2002) | 500 | 64.8 (2.1) | Cross-sectional |  |
| Manne-Goehler et al. (2017) | South Africa | The aim is assess the relationship between ART use and utilization of healthcare services for diabetes and hypertension. | HAALSI | 5059 | 63.6 (NR) | Longitudinal |  |
| Manne-Goehler et al. (2019) | Uganda | The aim of the study was to characterize associations between depression symptom severity and HIV infection, both prior to and years after ART initiation | Ugandan Non-Communicable Diseases & Aging Cohort Study | 296 | 51.9 (NR) | Longitudinal |  |
| Marcus et al. (2021) | South Africa | This study sought to evaluate the comparative effectiveness of three different home-based HIV testing strategies for older adults in rural South Africa. | HAALSI | 2963 | NR | Cross-sectional |  |
| Martinez et al. (2014) | South Africa | To determine if quality of life and social engagement varied across different drinking patterns among older south African adults | WHO SAGE | 3047 | 61.9 (NR) | Longitudinal |  |
| Maritz et al. (2018) | South Africa | To determine which biomarkers (metabolic, inflammatory, endothelial activation and oxidative stress) relate to aortic stiffness in young and older black South Africans, self-reporting no alcohol-use | PURE-SA-NWP | 322 | 61.6 (9.8) | Both |  |
| Matlho et al. (2019) | Botswana | This study examines HIV prevalence and related behaviors of this older cohort (50–64 years) in Botswana relative to the younger (25–49 years) cohort to better understand the challenges posed by this growing yet under-acknowledged group | Botswana AIDS Impact Survey IV | 5118 | NR | Cross-sectional |  |
| McKinnon et al. (2013) | Burkina Faso, Chad, Congo, Cote d’Ivoire, Ethiopia, Ghana, Kenya, Malawi, Mali, Namibia, Senegal, South Africa, Swaziland, Zambia, and Zimbabwe | To examine the relationship of living arrangement and depressive symptoms among older adults in Sub-Saharan Africa | World Health Surveys | 12647 | 62.1 (NR) | Cross-sectional |  |
| Menyanu et al. (2017) | Ghana and South Africa | To examines salt-related knowledge, attitude and self-reported behaviors (KAB) amongst adults from two African countries—Ghana and South Africa—which have distributed different public health messages related to salt | WHO SAGE | 10522 | NR | Cross-sectional |  |
| Minicuci et al. (2014) | Ghana | To generate results that help drive the issue of chronic diseases onto the agenda of the Ministry of Health/Ghana Health Service and push forward the implementation of the National Ageing Policy by the Ministry of Employment and Social Welfare | WHO SAGE | 4724 | 58.1 (NR) | Cross-sectional |  |
| Moore et al. (2018) | South Africa | To examine the multidimensional approach of social network and social determinants of health and self-reported physical health among older adults in Agincourt, South Africa. | HAALSI | 5059 | 62.4 (13.1) | Cross-sectional |  |
| Moreno-Agostino et al. (2020) | China, Ghana, India, Mexico, South Africa, Finland, Poland, and Spain | This study aims to determine whether there are differences in experiential wellbeing between retired and working older adults; whether time use accounts for a portion of these differences; and whether these potential relationships differ across LAMICs and high-income countries. | HAALSI | 29818 | 64.8 (10.5) | Longitudinal |  |
| Mtowa et al. (2017) | Tanzania | To estimate socio-demographic inequalities in HIV testing behavior and HIV prevalence among adults aged 50+ years, living in Ifakara town, Tanzania. | DSS-Tanzania | 13000 | 62.6 (2.2) | Cross-sectional |  |
| Mugisha et al. (2015) | Uganda | To examine gender roles in the provision and receipt of care among older Ugandans. | SAGE WOPS | 510 | 73.7 (1.1) | Cross-sectional |  |
| Mugisha et al. (2016) | Uganda | To describe the prevalence of chronic conditions and their risk factors and 2) to draw attention to associations between chronic conditions and disability | SAGE WOPS | 471 | 62.6 (1.2) | Cross-sectional |  |
| Mugisha et al. (2017) | Uganda | To document factors associated with the recency of health-care service utilization by people aged 50 years and over living with and without HIV in Uganda | SAGE WOPS | 510 | NR | Cross-sectional |  |
| Mugisha et al. (2018) | Uganda | To examine the association between social engagement and survival in people with or without HIV aged 50 years and over in Uganda | SAGE WOPS | 345 | 73.7 (1.1) | Cross-sectional |  |
| Mugisha et al. (2020) | Uganda | To document the survival rates of people aged 50+ years by HIV and treatment status | SAGE WOPS | 623 | NR | Cross-sectional |  |
| Mwanyangala et al. (2010) | Tanzania | To describe the impacts of ageing on the health status, quality of life and well-being of older people in a rural population of Tanzania | DSS-Tanzania | 5131 | 62.6 (9.2) | Longitudinal |  |
| Myroniuk (2017) | Malawi | To assess the marital dissolutions and the health of older adults in rural African contexts | MLSFH | 1200 | 59.6 (11.2) | Cross-sectional |  |
| Negin et al. (2010) | Kenya | To examine mortality due to AIDS in people aged 50 and older in an area of rural Kenya with high rates of HIV infection | MVP | 1228 | 61.6 (2.2) | Cross-sectional |  |
| Negin et al. (2011) | Malawi, Rwanda and Tanzania | To examine the prevalence of non-communicable disease (NCD) risk factors in rural Africa | MVP | 665 | 59.3 (8.0) | Cross-sectional |  |
| Negin et al. (2012a) | South Africa | To examine HIV awareness, attitudes, behavior and testing among older adults across a number of rural sites in Africa. | MVP | 1539 | 59.3 (8.0) | Cross-sectional |  |
| Negin et al. (2012b) | South Africa | To examine the prevalence of HIV and chronic comorbidities among those aged 50 years and older in South Africa using nationally representative data | WHO SAGE | 4227 | NR | Cross-sectional |  |
| Negin et al. (2016) | Uganda | To explore sexual behavior among older adults living with HIV in Uganda | SAGE WOPS | 101 | 61.0 (7.8) | Longitudinal |  |
| Negin et al. (2017) | South Africa | To examine the burden of HIV is increasing among adults aged over 50, who generally experience increased risk of comorbid illnesses and poorer financial protection. | WHO SAGE | 2872 | 61.5 (6.1) | Cross-sectional |  |
| Nikolov et al. (2020) | South Africa | To estimate and demonstrate the importance of specific cognitive domains in the classical Mincer equation. | HAALSI, CAPS | 9752 | NR | Cross-sectional |  |
| Nwakasi et al. (2019) | Ghana | To investigate some of the issues that may influence outpatient care utilization rate among older Ghanaians. | WHO SAGE | 5110 | 71.3 (2.1) | Cross-sectional |  |
| Nyirenda et al. (2012) | South Africa | To examine correlates of health and well-being of HIV-infected older people relative to HIV-affected people in rural South Africa | WHO SAGE | 5110 | 63.1 (2.1) | Cross-sectional |  |
| Nyirenda et al. (2013) | Uganda and South Africa | To describe and compare the health status, emotional wellbeing, and functional status of older people in Uganda and South Africa who are HIV infected or affected by HIV in their families | SAGE WOPS | 932 | 63.2 (2.0) | Cross-sectional |  |
| Ogunniyi et al. (2005) | Nigeria, USA | To investigated for the socio-demographic and self-reported medical as well as life-style risk factors in the cohorts as a way of explaining the site differences in AD incidence rates. | IIDP | 1255 | 77.4 (6.4) | Cross-sectional |  |
| Ojagbemi et al. (2013) | Nigeria | To report on a large community-based study of suicidal behavior among elderly persons in Nigeria. | ISA | 2152 | NR | Cross-sectional |  |
| Ojagbemi et al. (2015) | Nigeria | To investigate the relationship between gait speed and cognitive decline over 2 years in a community dwelling sample of elderly Africans. | ISA | 1042 | NR | Longitudinal |  |
| Ojagbemi et al. (2016) | Nigeria | To describe factors associated with incident dementia and dementia mortality over 5 years in a large community sample of elderly persons. | ISA | 1894 | 74.4 (8.8) | Longitudinal |  |
| Ojagbemi et al. (2017a) | Nigeria | To investigate the association of chronic conditions with incident and persistent disability among community-dwelling elderly Nigerians. | ISA | 1237 | 74.4 (8.8) | Longitudinal |  |
| Ojagbemi et al. (2017b) | Nigeria | To report on the impact of low socioeconomic position (SEP) on mortality over a 5-year observation period among community-dwelling older adults living in southwestern Nigeria. | ISA | 957 | 74.4 (8.8) | Longitudinal |  |
| Ojagbemi et al. (2018) | Nigeria | To investigate factors associated with sustained symptomatic remission (SR) from MDD and the 5-year trajectory of post-Major Depressive Disorder physical functioning | ISA | 201 | 74.2 (8.2) | Longitudinal |  |
| Oladeji et al. (2011) | Nigeria | To ascertain whether there is a differential risk of depression among persons with pain in different anatomical sites and to determine which pain conditions are independent risk factors for depression. | ISA | 2152 | NR | Cross-sectional |  |
| Payne et al. (2013) | Malawi | To investigate how poor physical health results in functional limitations that limit the day-to-day activities of individuals in domains relevant to this subsistence-agriculture context | MLSFH | 1075 | NR | Longitudinal |  |
| Payne et al. (2017a) | South Africa | To describe the prevalence and correlates of phenotypic frailty using data from the Health and Aging in Africa: Longitudinal Studies of an INDEPTH Community cohort. | HAALSI | 5059 | NR | Cross-sectional |  |
| Payne et al. (2017b) | South Africa | To describe physical functioning in this aging population and place the overall level and age-trajectories of physical health in the context of other Health and Retirement Study (HRS) sister studies in low- and middle-income countries (LMICs). | HAALSI | 5059 | NR | Cross-sectional |  |
| Payne et al. (2020) | South Africa | To investigate the relationships between exposure to life-course traumatic events (TEs) and later life mental, physical, and cognitive health outcomes in the older population of a rural South African community. | HAALSI | 2473 | NR | Longitudinal |  |
| Peltzer & Pengpid (2018) | South Africa | To investigate sleep duration and its association with sociodemographic, health behavior, mental health, and chronic disease factors among rural individuals 40 years and older in South Africa. | WHO SAGE | 4725 | 61.5 (13.0) | Cross-sectional |  |
| Peltzer & Phaswana-Mafuya (2012a) | South Africa | To evaluate the degree of perceived responsiveness with outpatient and inpatient healthcare in South Africa. | WHO SAGE | 3840 | 61.6 (NR) | Cross-sectional |  |
| Peltzer & Phaswana-Mafuya (2012b) | South Africa | To determine the prevalence of tobacco use and their associated factors in older South Africans | WHO SAGE | 3840 | 61.6 (NR) | Cross-sectional |  |
| Peltzer & Phaswana-Mafuya (2012c) | South Africa | To investigate the prevalence and associated factors of low fruit and vegetable consumption in a national probability sample of older South Africans who participated in the Study of Global Ageing and Adults Health (SAGE) in 2008. | WHO SAGE | 3840 | 61.6 (NR) | Cross-sectional |  |
| Peltzer & Phaswana-Mafuya (2013a) | South Africa | To investigate the prevalence and associated factors of self-reported symptom-based depression in a national sample of older South Africans who participated in the Study of Global Ageing and Adult Health (SAGE wave 1) in 2008. | WHO SAGE | 3840 | NR | Cross-sectional |  |
| Peltzer & Phaswana-Mafuya (2013b) | South Africa | To investigate the prevalence and associated factors of hypertension in a national sample of older South Africans who participated in the Study of Global Ageing and Adults’ Health (SAGE) in 2008. | WHO SAGE | 3840 | NR | Cross-sectional |  |
| Peltzer & Phaswana-Mafuya (2013c) | South Africa | To assess the prevalence and to identify the factors associated with arthritis in a national probability sample of older South Africans who participated in the Study of Global Ageing and Adults Health (SAGE) in 2008 | WHO SAGE | 3840 | NR | Cross-sectional |  |
| Peltzer & Phaswana-Mafuya (2014) | South Africa | To establish their prevalence estimates and correlates among older South African women who participated in the Study of Global Ageing and Adults Health (SAGE) in 2008 | WHO SAGE | 3840 | NR | Cross-sectional |  |
| Peltzer & Phaswana-Mafuya (2017) | South Africa | To estimate the association between visual impairment and low vision and sleep duration and poor sleep quality in a national sample of older adults in South Africa | WHO SAGE | 3840 | NR | Cross-sectional |  |
| Peltzer (2017) | South Africa | To investigate sleep duration in four different population groups in a national probability sample of older South Africans who participated in the Study of Global Ageing and Adult Health (SAGE) Wave 1 | WHO SAGE | 3284 | 62.1 (NR) | Cross-sectional |  |
| Pengpid & Peltzer (2018) | South Africa | To examine prevalence and social and health correlates of insomnia symptoms among middle- and older-adults in rural South Africa | HAALSI | 5059 | 62.4 (NR) | Cross-sectional |  |
| Pengpid & Peltzer (2019) | South Africa | To assess the association of sedentary behavior with 12 different sleep problem indicators among rural middle-aged and elderly adults in South Africa | HAALSI | 47782 | NR | Cross-sectional |  |
| Pengpid & Peltzer (2020) | South Africa | To investigate the relationship between poor mental health and socio-demographic factors, health, and chronic conditions among rural middle-older persons in South Africa | HAALSI | 5059 | 63.1 (NR) | Longitudinal |  |
| Phaswana-Mafuya & Peltzer (2018) | South Africa | To assess ethnic health disparities in four elderly population groups. | WHO SAGE | 3284 | NR | Cross-sectional |  |
| Phaswana-Mafuya et al. (2013a) | South Africa | To explore the sociodemographic predictors of multiple non-communicable disease (NCD) risk factors experienced by elderly South Africans. | WHO SAGE | 3840 | NR | Cross-sectional |  |
| Phaswana-Mafuya et al. (2013b) | South Africa | To investigate the self-reported prevalences of major chronic NCDs and their predictors among older South Africans. | WHO SAGE | 3840 | NR | Cross-sectional |  |
| Putnam et al. (2018) | Tanzania | To establish the effect of hypertension at the organ level by measuring the prevalence of EOD in a cohort of community-dwelling older adults living in rural Tanzania. | DSS-Tanzania | 246 | NR | Cross-sectional |  |
| Raji et al. (2017) | Nigeria | To determine the prevalence, awareness, treatment and control of hypertension among the study population. | ISA | 2152 | 76.9 (8.4) | Cross-sectional |  |
| Ralston et al. (2016) | South Africa | To address these issues through the following questions: (a) Are pension recipients better off than eligible non-recipients? (b)What household and individual characteristic are associated with pension receipt? | DSS-Agincourt, WHO SAGE | 4915 | NR | Cross-sectional |  |
| Ralston et al. (2022) | South Africa | To fill these gaps by exploring the relationships between specific types of social support and physical health in baseline data (2014–2015) from the Health and Aging in Africa | HAALSI | 6281 | NR | Cross-sectional |  |
| Ralston (2018) | South Africa | To evaluate the influence of local district conditions on subjective quality of life of older South African adults | WHO SAGE | 2937 | NR | Cross-sectional |  |
| Ramlagan et al. (2013) | South Africa | To investigate the association between social capital and several health variables, namely: self-rated health, depressive symptoms, cognitive functioning and physical inactivity, among older South Africans. | WHO SAGE | 3840 | 50+ | Cross-sectional |  |
| Ramlagan et al. (2014) | South Africa | To investigate the social and health differences in hand grip strength among older adults in a national probability sample of older South Africans who participated in the Study of Global Ageing and Adults Health (SAGE wave 1) in 2008 | WHO SAGE | 3840 | 61.6 (9.5) | Cross-sectional |  |
| Randall & Coast (2016) | Not specified (used DHS across 17 countries) | To evaluate the quality of nationally representative data on older Africans through examining the accuracy of age data collected from different sources | Demographic and Health Surveys (DHS), (b) Census data (c) Living Standards Measurement Study (LSMS) | 32954 | NR | Cross-sectional |  |
| Reiger et al. (2017) | South Africa | To assess the prevalence, awareness, treatment, and control of dyslipidemia in rural South Africa and how they are impacted by different behaviors and non-modifiable factors | HAALSI | 4247 | 61.9 (12.9) | Cross-sectional |  |
| Rishworth et al. (2020) | Uganda | To examine the (i) direct and indirect efects of age on subjective wellbeing (SWB) through social and structural determinants, and (ii) how direct and indirect e ects vary by gender. | SAGE WOPS | 470 | 65.0 (10.3) | Cross-sectional |  |
| Rohr et al. (2017) | South Africa | To evaluate the accuracy of self-reported HIV status, which may provide useful information for targeting interventions or offer an alternative to biomarker testing | HAALSI | 4560 | NR | Cross-sectional |  |
| Rohr et al. (2020) | South Africa | We use both biomarkers and self-reported data from a large population-based cohort of older South Africans to establish the first HIV cascade for this growing segment of the HIV-positive population and compare results using the different data sources | HAALSI | 5059 | NR | Cross-sectional |  |
| Rosenberg et al. (2017) | South Africa | To identify unmet needs for HIV prevention among older adults in rural south Africa | HAALSI | 5059 | NR | Cross-sectional |  |
| Rosenberg et al. (2018) | South Africa | The study examined how HIV prevalence differs by circumcision status in older adult men in a rural South African community, a non-experimental setting in a time of expanding VMMC access | HAALSI & DSS-Agincourt | 2345 | NR | Cross-sectional |  |
| Rosenberg et al. (2020) | South Africa | To assess the independent relationships among each of cognitive function, literacy and education with HIV status knowledge in a population‐based sample of older adults living in a rural South African community with high HIV prevalence. | HAALSI | 5059 | NR | Cross-sectional |  |
| Rossouw & Smith (2017) | South Africa | To explore the association between education levels and reporting behavior in terms of Health-system responsiveness (HSR) in South Africa | WHO SAGE | 1499 | 62.5 (9.0) | Cross-sectional |  |
| Saeed et al. (2016) | Ghana | To investigate the effect of socio-economic inequality in the use of healthcare services among older adult men and women in Ghana | WHO SAGE | 5573 | NR | Cross-sectional |  |
| Samba et al. (2019) | Republic of Congo | To assess the prognostic value of the Ankle Brachial Index (ABI) measurement as a screening tool for Peripheral artery disease (PAD), to predict mortality risk among Congolese older adults. | EPIDEMCA-FU (Epidemiology of Dementia in Central Africa - Follow-Up) | 1029 | 73.8 (6.8) | Longitudinal |  |
| Sanuade et al. (2019) | Ghana | To examine the prevalence and correlates of stroke among older adults in Ghana | WHO SAGE | 4279 | NR | Cross-sectional |  |
| Schafer et al. (2021) | South Africa | To examine if older adults with HIV have distinctive personal networks? stigma, network activation and it disclosure in south africa | HAALSI | 5059 | 69.0 (NR) | Cross-sectional |  |
| Schatz (2007) | South Africa | To examine financial, emotional, and physical responsibilities elderly women are being asked to take on due to the incapacity of their adult children to care for the next generation | HAALSI | 70272 | NR | Cross-sectional |  |
| Schatz et al. (2012) | South Africa | To explore the effect of the pension for health and wellbeing indicators of rural South African men and women | WHO SAGE | 4085 | NR | Cross-sectional |  |
| Schatz et al. (2015) | South Africa | To determine how have the living arrangements of elderly persons changed over three time points, namely in 2000, 2005 and 2010. To understand if the distribution of living arrangements vary by pension-eligibility status of the older person in the household. To determine the differences in household composition of those households where an older person is a dependent member versus those where he or she is a productive member? | DSS-Agincourt | 19384 | NR | Longitudinal |  |
| Schatz et al. (2018) | South Africa | To explore older persons’ self-reported disability by living arrangements and gender, paying particular attention to various multigenerational arrangements. | DSS-Agincourt | 6730 | NR | Cross-sectional |  |
| Scholten et al. (2011) | Uganda | To describe health among older people in association with the HIV epidemic | The Entebbe HIV cohort | 510 | NR | Cross-sectional |  |
| Stringhini et al. (2018) | South Africa and Ghana | To assess the association of low socioeconomic status and risk factors for non-communicable diseases (diabetes, high alcohol intake, high blood pressure, obesity, physical inactivity, smoking) with loss of physical functioning at older ages. | HAALSI, WHO SAGE | 8846 | Women: 63.6 (9.8); Men: 63.9 (9.4) | Cross-sectional |  |
| Tomaz et al. (2020) | South Africa | To examine self-reported physical activity in middle-aged and older adults in rural south-africa: levels an correlates | HAALSI | 5059 | 69.0 (NR) | Cross-sectional |  |
| van Empel et al. (2021) | South Africa | To explore the perceptions of HIV acquisition risk and prevalence among older adults in rural South Africa | HAALSI | 5059 | 40+ | Cross-sectional |  |
| Wade et al. (2021a) | South Africa | To investigated concordance between hemoglobin A1c (HbA1c)-defined diabetes and fasting plasma glucose (FPG)-defined diabetes in a black South African population with a high prevalence of obesity. | HAALSI | 765 | 55.0 (NR) | Cross-sectional |  |
| Wade et al. (2021b) | South Africa | To investigate the relationship between multimorbidity (≥2 of the following chronic medical conditions: hypertension, diabetes, dyslipidemia, anemia, HIV, angina, depression, post-traumatic stress disorder, alcohol dependence) and all-cause mortality in an older, rural black South African population. | HAALSI | 4455 | 61.0 (NR) | Longitudinal |  |
| Wagner et al. (2018) | South Africa | To investigate overall and sex-specific determinants of BMI in a rural adult South African population undergoing rapid social and epidemiological transitions. | HAALSI | 2234 | NR | Longitudinal |  |
| Wallrauch et al. (2010) | South Africa | To determine the HIV prevalence and incidence in people 50 years and older in rural South Africa. | ACDIS | 2791 | 50+ | Longitudinal |  |
| Ware et al. (2017) | South Africa | To explore the relationship between salt and blood pressure (BP) in a subsample of the World Health Organization Study on global AGEing and adult health (SAGE) Wave 2 before implementation of legislation in South Africa. | WHO SAGE | 2722 | 55.0 (21.0) | Longitudinal |  |
| Waterhouse et al. (2017) | South Africa | To investigate the association between multi-morbidity and disability among older adults; and second, to examine whether hypertension (both diagnosed and undiagnosed) mediates this relationship. Lastly, we consider whether the impact of the multi-morbidity on disability varies by socio-demographic characteristics. | WHO SAGE | 3842 | NR | Cross-sectional |  |
| Watkins et al. (2014) | South Africa | To profile the economic impact of CVD care on South African individuals and their households | WHO SAGE | 4895 | NR | Cross-sectional |  |
| Wilunda et al. (2015) | Kenya | To improve understanding of functional health and well-being in older adult slum-dwellers in Nairobi (Kenya). | DSS-Nairobi, WHO SAGE | 1878 | 50+ | Cross-sectional |  |
| Yawson et al. (2013) | Ghana | To describes demographic, socioeconomic, health risks and life satisfaction indices related to tobacco use among older adults in Ghana. | WHO SAGE | 4252 | 64.4 (2.1) | Cross-sectional |  |
| Yawson et al. (2014) | Ghana | To describes cataract, a chronic eye condition, self-reported among older adults in Ghana and the need for improving access to eye care services. | WHO SAGE | 4278 | 64.4 (2.4) | Cross-sectional |  |
| Yoro-Zohoun et al. (2019) | Central African Republic and the Republic of the Congo | To describe the severity of neuropsychiatric symptoms among older people, evaluate the distress experienced by caregivers, and assess which neuropsychiatric symptoms were specifically associated with dementia among older adults in Central Africa. | EPIDEMCA | 532 | NR | Cross-sectional |  |
| Yoro-Zohoun et al. (2021) | Central African Republic and the Republic of the Congo | To evaluate the association between neuropsychiatric symptoms and apolipoprotein E (APOE) ϵ4 allele among older people in Central African Republic (CAR) and the Republic of Congo (ROC). | EPIDEMCA | 322 | NR | Cross-sectional |  |
| Zengin et al. (2017) | Gambia | To Investigate bone and muscle ageing in men and women from a poor, subsistence farming community of The Gambia, West Africa | GamBAS | 488 | NR | Longitudinal |  |
| Zengin et al. (2018) | Gambia | To investigate the prevalence of sarcopenia, assessed the suitability of current diagnostic guidelines and explored muscle–bone relationships in ageing men and women from rural Gambia. | GamBAS | 488 | NR | Cross-sectional |  |
| **Note:** **ACDIS** = Africa Centre Demographic Information System; **CAPS** = Cape Area Panel Study; **DSS** = Demographic Surveillance System; **EPIDEMICA** = Epidemiology of Dementia in Central Africa; **GamBAS** = Gambian Bone and Muscle Aging Study; **HAALSI** = Health and Aging in Africa: A Longitudinal Study of an INDEPTH Community in South Africa; **ISA** = Ibadan Study of Aging; **IIDP** = Indianapolis-Ibadan Dementia Project; **ICAP** = International Center for AIDS Care and Treatment Program, Columbia University; **KHDS** = Kagera Health and Development Survey; **MLSFH** = Malawi Longitudinal Study of Families and Health; **MVP** = Millennium Villages Project; **PURE-SA-NWP** = South African leg of the international Prospective Urban and Rural Epidemiology study; **UPHD** = Urbanization Poverty and Health Dynamics; **WHO SAGE** = World Health Organization - Study on global AGEing and adult health. | | | | | | | |

**Reference list of all included articles(n=193)**

Ackuaku-Dogbe, E. M., Yawson, A. E., & Biritwum, R. B. (2015). Cataract surgical uptake among older adults in Ghana. *Ghana medical journal*, *49*(2), 84-89.

Adams, L. B., Farrell, M., Mall, S., Mahlalela, N., & Berkman, L. (2020). Dimensionality and differential item endorsement of depressive symptoms among aging Black populations in South Africa: Findings from the HAALSI study. *Journal of affective disorders*, *277*, 850-856.

Adhvaryu, A. R., & Beegle, K. (2012). To provide empirical evidence of the impacts of adult deaths on older adults in Kagera, northwest Tanzania. *Economic development and cultural change, 60*(2), 245–277. <https://doi.org/10.1086/662577>

Aheto, J. M. K., Udofia, E. A., Kallson, E., Mensah, G., Nadia, M., Nirmala, N., ... & Yawson, A. E. (2020). Prevalence, socio-demographic and environmental determinants of asthma in 4621 Ghanaian adults: Evidence from Wave 2 of the World Health Organization’s study on global AGEing and adult health. *Plos one*, *15*(12), e0243642.

Akuamoah-Boateng, H. (2013). Self-reported vision health status among older people in the Kassena-Nankana District, Ghana. *Global health action*, *6*(1), 19012.

Amegbor, P. M., Kuuire, V. Z., Robertson, H., & Kuffuor, O. A. (2018). Predictors of basic self-care and intermediate self-care functional disabilities among older adults in Ghana. *Archives of gerontology and geriatrics*, *77*, 81-88.

Amegbor, P. M., Braimah, J. A., Adjaye-Gbewonyo, D., Rosenberg, M. W., & Sabel, C. E. (2020). Effect of cognitive and structural social capital on depression among older adults in Ghana: A multilevel cross-sectional analysis. *Archives of Gerontology and Geriatrics*, *89*, 104045.

Amegbor, P. M., Kuuire, V. Z., Yawson, A. E., Rosenberg, M. W., & Sabel, C. E. (2021). Social frailty and depression among older adults in Ghana: insights from the WHO SAGE Surveys. *Research on Aging*, *43*(2), 85-95.

Amegbor, P. M., & Rosenberg, M. W. (2022). Health and socioeconomic risk factors for overnight admission among older adults in Ghana. *Journal of Population Ageing*, *15*(4), 961-979.

Annin, K., Saeed, B. I., Yawson, A., Musah, A. A. I., Nakua, E., Agyei-Baffour, P., & Nsowah-Nuamah, N. N. N. (2014). Assessing the association between the degree of pain and socioeconomic status among older persons in Ghana. *Global journal of health science*, *6*(3), 155.

Ardington, C., Case, A., Islam, M., Lam, D., Leibbrandt, M., Menendez, A., & Olgiati, A. (2010). The impact of AIDS on intergenerational support in South Africa: Evidence from the cape area panel study. *Research on aging, 32*(1), 97–121. <https://doi.org/10.1177/0164027509348143>

Asiimwe, S. B., Farrell, M., Kobayashi, L. C., Manne-Goehler, J., Kahn, K., Tollman, S. M., ... & Bärnighausen, T. (2020). Cognitive differences associated with HIV serostatus and antiretroviral therapy use in a population-based sample of older adults in South Africa. *Scientific Reports*, *10*(1), 16625.

Awoke, M. A., Negin, J., Moller, J., Farell, P., Yawson, A. E., Biritwum, R. B., & Kowal, P. (2017). Predictors of public and private healthcare utilization and associated health system responsiveness among older adults in Ghana. *Global health action, 10*(1). <https://doi.org/10.1080/16549716.2017.1301723>

Ayernor, P. K. (2012). Diseases of ageing in Ghana. *Ghana medical journal, 46*(2 Suppl), 18–22.

Barker, F. J., Davies, J. I., Gomez-Olive, F. X., Kahn, K., Matthews, F. E., Payne, C. F., ... & Witham, M. D. (2021). Developing and evaluating a frailty index for older South Africans—findings from the HAALSI study. *Age and ageing*, *50*(6), 2167-2173.

Balogun, S., Yusuff, H., Adeleye, B., Balogun, M., Aminu, A., Yusuf, K., & Tettey, P. (2018). Determinants of bed net use among older people in Nigeria: results from a nationally representative survey. *Pan African Medical Journal*, *31*(1).

Bastawrous, A., Mathenge, W., Wing, K., Rono, H., Gichangi, M., Weiss, H. A., Macleod, D., Foster, A., Burton, M. J., & Kuper, H. (2016). Six-year incidence of blindness and visual impairment in Kenya: The Nakuru eye disease cohort study. *Investigative ophthalmology and visual science, 57*(14), 5974–5983. <https://doi.org/10.1167/iovs.16-19835>

Baiyewu, O., Smith-Gamble, V., Lane, K. A., Gureje, O., Gao, S., Ogunniyi, A., ... & Hendrie, H. C. (2007). Prevalence estimates of depression in elderly community-dwelling African Americans in Indianapolis and Yoruba in Ibadan, Nigeria. *International psychogeriatrics*, *19*(4), 679-689.

Baiyewu, O., Unverzagt, F. W., Ogunniyi, A., Smith‐Gamble, V., Gureje, O., Lane, K. A., ... & Hendrie, H. C. (2012). Behavioral symptoms in community‐dwelling elderly Nigerians with dementia, mild cognitive impairment, and normal cognition. *International journal of geriatric psychiatry*, *27*(9), 931-939.

Bekibele, C. O., & Gureje, O. (2010). Fall incidence in a population of elderly persons in Nigeria. *Gerontology*, *56*(3), 278-283.

Bennett, R., Chepngeno-Langat, G., Evandrou, M., & Falkingham, J. (2016). Gender differentials and old age survival in the Nairobi slums, Kenya. *Social Science & Medicine*, *163*, 107-116.

Biritwum, R. B., Mensah, G., Minicuci, N., Yawson, A. E., Naidoo, N., Chatterji, S., & Kowal, P. (2013). Household characteristics for older adults and study background from SAGE Ghana Wave 1. *Global health action, 6*(1). <https://doi.org/10.3402/gha.v6i0.20096>

Boateng, G. O., Adams, E. A., Odei Boateng, M., Luginaah, I. N., & Taabazuing, M. M. (2017). Obesity and the burden of health risks among the elderly in Ghana: A population study. *PloS one, 12*(11), e0186947. <https://doi.org/10.1371/journal.pone.0186947>

Calys-Tagoe, B. N. L., Hewlett, S. A., Dako-Gyeke, P., Yawson, A. E., Baddoo, N. A., Seneadza, N. A. H., Mensah, G., Minicuci, N., Naidoo, N., Chatterji, S., Kowal, P., & Biritwum, R. B. (2014). Predictors of subjective well-being among older Ghanaians. *Ghana medical journal, 48*(4), 178–184. <https://doi.org/10.4314/gmj.v48i4.2>

Calys-Tagoe, B., Nuertey, B. D., Tetteh, J., & Yawson, A. E. (2020). Individual awareness and treatment effectiveness of hypertension among older adults in Ghana: evidence from the World Health Organization study of global ageing and adult health wave 2. *The pan African medical journal*, 37.

Chang, A. Y., Gómez-Olivé, F. X., Manne-Goehler, J., Wade, A. N., Tollman, S., Gaziano, T. A., & Salomon, J. A. (2019a). Multimorbidity and care for hypertension, diabetes and HIV among older adults in rural South Africa. *Bulletin of the World Health Organization*, *97*(1), 10.

Chang, A. Y., Gómez-Olivé, F. X., Payne, C., Rohr, J. K., Manne-Goehler, J., Wade, A. N., ... & Salomon, J. A. (2019b). Chronic multimorbidity among older adults in rural South Africa. *BMJ global health*, *4*(4), e001386.

Charlton, K. E., Schutte, A. E., Wepener, L., Corso, B., Kowal, P., & Ware, L. J. (2020). Correcting for intra-individual variability in sodium excretion in spot urine samples does not improve the ability to predict 24h urinary sodium excretion. *Nutrients, 12*(7), 2026. <https://doi.org/10.3390%2Fnu12072026>

Chepngeno-Langat, G. (2013). Perception of vulnerability to HIV infection among older people in Nairobi, Kenya: a need for intervention. *Journal of biosocial science*, *45*(2), 249-266.

Chepngeno‐Langat, G. (2014). Entry and re‐entry into informal care‐giving over a 3‐year prospective study among older people in N airobi slums, K enya. *Health & Social Care in the Community*, *22*(5), 533-544.

Chepngeno-Langat, G., Madise, N., Evandrou, M., & Falkingham, J. (2011). Gender differentials on the health consequences of care-giving to people with AIDS-related illness among older informal carers in two slums in Nairobi, Kenya. *AIDS care, 23*(12), 1586–1594. <https://doi.org/10.1080/09540121.2011.569698>

Chepngeno‐Langat, G., Falkingham, J. C., Madise, N. J., & Evandrou, M. (2012). Concern about HIV and AIDS among older people in the slums of Nairobi, Kenya. *Risk Analysis: An International Journal*, *32*(9), 1512-1523.

Chepngeno-Langat, G., Van Der Wielen, N., Evandrou, M., & Falkingham, J. (2019). Unravelling the wider benefits of social pensions: Secondary beneficiaries of the older persons cash transfer program in the slums of Nairobi. *Journal of aging studies, 51*, 100818.

Clark, D. O., Gao, S., Lane, K. A., Callahan, C. M., Baiyewu, O., Ogunniyi, A., & Hendrie, H. C. (2014). Obesity and 10-year mortality in very old african americans and yoruba-nigerians: Exploring the obesity paradox. *Journals of gerontology - series A biological sciences and medical sciences, 69*(9), 1162–1169. <https://doi.org/10.1093/gerona/glu035>

Dei, V., & Sebastian, M. S. (2018). Is healthcare really equal for all? Assessing the horizontal and vertical equity in healthcare utilisation among older Ghanaians. *International journal for equity in health, 17*(1), 86. <https://doi.org/10.1186/s12939-018-0791-3>

Eduardo, Eduard, Matthew R. Lamb, Sasi Kandula, Andrea Howard, Veronicah Mugisha, Davies Kimanga, Bonita Kilama, Wafaa El-Sadr, and Batya Elul. "Characteristics and outcomes among older HIV-positive adults enrolled in HIV programs in four sub-Saharan African countries." *PloS one* 9, no. 7 (2014): e103864.

Fantahun, M., Berhane, Y., Högberg, U., Wall, S., & Byass, P. (2009). Ageing of a rural Ethiopian population: who are the survivors?. *Public Health*, *123*(4), 326-330.

Ferrari, R., Ford, I., Greenlaw, N., Tardif, J. C., Tendera, M., Abergel, H., Fox, K., Hu, D., Shalnova, S., Steg, P. G., & CLARIFY Registry Investigators (2015). Geographical variations in the prevalence and management of cardiovascular risk factors in outpatients with CAD: Data from the contemporary CLARIFY registry. *European journal of preventive cardiology, 22*(8), 1056–1065. <https://doi.org/10.1177/2047487314547652>

Farrell, M. T., Kobayashi, L. C., Montana, L., Wagner, R. G., Demeyere, N., & Berkman, L. (2020). Disparity in educational attainment partially explains cognitive gender differences in older rural South Africans. *The Journals of Gerontology: Series B*, *75*(7), e161-e173.

Ferro, E. G., Abrahams-Gessel, S., Jardim, T. V., Wagner, R., Gomez-Olive, F. X., Wade, A. N., ... & Gaziano, T. A. (2021). Echocardiographic and electrocardiographic abnormalities among elderly adults with cardiovascular disease in rural South Africa. *Circulation: Cardiovascular Quality and Outcomes*, *14*(11), e007847.

Gao, S., Ogunniyi, A., Hall, K. S., Baiyewu, O., Unverzagt, F. W., Lane, K. A., ... & Hendrie, H. C. (2016). Dementia incidence declined in African-Americans but not in Yoruba. *Alzheimer's & Dementia*, *12*(3), 244-251.

Gatimu, S. M., Milimo, B. W., & Sebastian, M. S. (2016). Prevalence and determinants of diabetes among older adults in Ghana. *BMC public health*, *16*(1), 1-12.

Gaziano, T. A., Abrahams-Gessel, S., Gomez-Olive, F. X., Wade, A., Crowther, N. J., Alam, S., ... & Tollman, S. (2017). Cardiometabolic risk in a population of older adults with multiple co-morbidities in rural south africa: the HAALSI (Health and Aging in Africa: longitudinal studies of INDEPTH communities) study. *BMC Public Health*, *17*(1), 1-10.

Geldsetzer, P., Vaikath, M., Wagner, R., Rohr, J. K., Montana, L., Gómez-Olivé, F. X., ... & Berkman, L. F. (2019). Depressive symptoms and their relation to age and chronic diseases among middle-aged and older adults in rural South Africa. *The Journals of Gerontology: Series A*, *74*(6), 957-963.

Gildner, T. E., Liebert, M. A., Kowal, P., Chatterji, S., & Josh Snodgrass, J. (2014). Sleep duration, sleep quality, and obesity risk among older adults from six middle‐income countries: Findings from the study on global AGEing and adult health (SAGE). *American Journal of Human Biology*, *26*(6), 803-812.

Gómez-Olivé, F. X., Rohr, J. K., Roden, L. C., Rae, D. E., & von Schantz, M. (2018). Associations between sleep parameters, non-communicable diseases, HIV status and medications in older, rural South Africans. *Scientific Reports*, *8*(1), 1-11.

Gómez-Olivé, X., Thorogood, M., Bocquier, P., Mee, P., Kahn, K., Berkman, L., & Tollman, S. (2014a). Social Conditions and Disability Related to the Mortality of Older People in Rural South Africa. *World health & population, 15*(4), 34–43. <https://doi.org/10.12927/whp.2015.24266>

Gómez-Olivé, F. X., Thorogood, M., Kandala, N. B., Tigbe, W., Kahn, K., Tollman, S., & Stranges, S. (2014b). Sleep problems and mortality in rural South Africa: novel evidence from a low-resource setting. *Sleep medicine*, *15*(1), 56-63.

Gómez-Olivé, F. X., Thorogood, M., Clark, B., Kahn, K., & Tollman, S. (2013). Self-reported health and health care use in an ageing population in the Agincourt sub-district of rural South Africa. *Global health action, 6*, 19305. <https://doi.org/10.3402/gha.v6i0.19305>

Gray, W. K., Dewhurst, F., Dewhurst, M. J., Orega, G., Kissima, J., Chaote, P., & Walker, R. W. (2016). Rates and predictors of three-year mortality in older people in rural Tanzania. *Archives of gerontology and geriatrics, 62*, 36–42. <https://doi.org/10.1016/j.archger.2015.10.008>

Gray, W. K., Orega, G., Kisoli, A., Rogathi, J., Paddick, S. M., Longdon, A. R., Walker, R. W., Dewhurst, F., Dewhurst, M., Chaote, P., & Dotchin, C. (2017). Identifying Frailty and its Outcomes in Older People in Rural Tanzania. *Experimental aging research, 43*(3), 257–273. <https://doi.org/10.1080/0361073X.2017.1298957>

Gureje, O., Ogunniyi, A., & Kola, L. (2006). The profile and impact of probable dementia in a sub-Saharan African community: results from the Ibadan Study of Aging. *Journal of psychosomatic research*, *61*(3), 327-333.

Gureje, O., Kola, L., & Afolabi, E. (2007). Epidemiology of major depressive disorder in elderly Nigerians in the Ibadan Study of Ageing: a community-based survey. *The Lancet*, *370*(9591), 957-964.

Gureje, O., Ogunniyi, A., Kola, L., & Afolabi, E. (2006). Functional disability in elderly Nigerians: Results from the Ibadan Study of Aging. *Journal of the American Geriatrics Society*, *54*(11), 1784-1789.

Gureje, O., Ogunniyi, A., Baiyewu, O., Price, B., Unverzagt, F. W., Evans, R. M., ... & Murrell, J. R. (2006). APOE ε4 is not associated with Alzheimer's disease in elderly Nigerians. *Annals of neurology*, *59*(1), 182-185.

Gureje, O., Ademola, A., & Olley, B. O. (2008). Depression and disability: comparisons with common physical conditions in the Ibadan study of aging. *Journal of the American Geriatrics Society*, *56*(11), 2033-2038.

Gureje, O., Kola, L., Afolabi, E., & Olley, B. O. (2008). Determinants of quality of life of elderly Nigerians: results from the Ibadan study of ageing. *African journal of medicine and medical sciences*, *37*(3), 239.

Gureje, O., Kola, L., Ademola, A., & Olley, B. O. (2009). Profile, comorbidity and impact of insomnia in the Ibadan study of ageing. *International Journal of Geriatric Psychiatry: A journal of the psychiatry of late life and allied sciences*, *24*(7), 686-693.

Gureje, O., Oladeji, B. D., Abiona, T., Makanjuola, V., & Esan, O. (2011a). The natural history of insomnia in the Ibadan study of ageing. *Sleep, 34*(7), 965–973. <https://doi.org/10.5665/SLEEP.1138>

Gureje, O., Ogunniyi, A., Kola, L., & Abiona, T. (2011b). Incidence of and risk factors for dementia in the Ibadan study of aging. *Journal of the american geriatrics society, 59*(5), 869–874. <https://doi.org/10.1111/j.1532-5415.2011.03374.x>

Gureje, O., Oladeji, B. D., Abiona, T., & Chatterji, S. (2014). Profile and determinants of successful aging in the Ibadan Study of Ageing. *Journal of the American Geriatrics Society*, *62*(5), 836-842.

Harling, G., Kobayashi, L. C., Farrell, M. T., Wagner, R. G., Tollman, S., & Berkman, L. (2020b). Social contact, social support, and cognitive health in a population-based study of middle-aged and older men and women in rural South Africa. *Social Science & Medicine*, *260*, 113167

Harling, G., Payne, C. F., Davies, J. I., Gomez-Olive, F. X., Kahn, K., Manderson, L., ... & Witham, M. D. (2020a). Impairment in activities of daily living, care receipt, and unmet needs in a middle-aged and older rural South African population: Findings from the HAALSI study. *Journal of aging and health*, *32*(5-6), 296-307.

Hall, K. 2., Murrell, J., Ogunniyi, A., Deeg, M., Baiyewu, O., Gao, S., ... & Hendrie, H. (2006). Cholesterol, APOE genotype, and Alzheimer disease: an epidemiologic study of Nigerian Yoruba. *Neurology*, *66*(2), 223-227.

Hendrie, H. C., Murrell, J., Baiyewu, O., Lane, K. A., Purnell, C., Ogunniyi, A., ... & Gao, S. (2014). APOE ε4 and the risk for Alzheimer disease and cognitive decline in African Americans and Yoruba. *International psychogeriatrics*, *26*(6), 977-985.

Hendrie, H. C., Hall, K. S., Ogunniyi, A., & Gao, S. (2004). Alzheimer's disease, genes, and environment: the value of international studies. *The Canadian Journal of Psychiatry*, *49*(2), 92-99.

Heyns, C. F., Fisher, M., Lecuona, A., & van der Merwe, A. (2011). Prostate cancer among different racial groups in the western cape: Presenting features and management. *South african medical journal, 101*(4), 267–270. <https://doi.org/10.7196/samj.4420>

Hosegood, V., & Timaeus, I. M. (2005). The impact of adult mortality on the living arrangements of older people in rural South Africa. *Ageing & Society*, *25*(6), 431-444.

Houle, B., Gaziano, T., Farrell, M., Gómez-Olivé, F. X., Kobayashi, L. C., Crowther, N. J., ... & Tollman, S. M. (2019). Cognitive function and cardiometabolic disease risk factors in rural South Africa: baseline evidence from the HAALSI study. *BMC Public Health*, *19*, 1-11.

Humphreys, G. W., Duta, M. D., Montana, L., Demeyere, N., McCrory, C., Rohr, J., ... & Berkman, L. (2017). Cognitive function in low-income and low-literacy settings: Validation of the tablet-based Oxford Cognitive Screen in the Health and Aging in Africa: A Longitudinal Study of an INDEPTH community in South Africa (HAALSI). *The Journals of Gerontology: Series B*, *72*(1), 38-50.

Ice, G. H., Yogo, J., Heh, V., & Juma, E. (2010). The impact of caregiving on the health and well-being of Kenyan Luo grandparents. *Research on Aging*, *32*(1), 40-66.

Jardim, T. V., Reiger, S., Abrahams-Gessel, S., Crowther, N. J., Wade, A., Gomez-Olive, F. X., ... & Gaziano, T. A. (2017). Disparities in Management of Cardiovascular Disease in Rural South Africa: Data From the HAALSI Study (Health and Aging in Africa: Longitudinal Studies of International Network for the Demographic Evaluation of Populations and Their Health Communities). *Circulation: Cardiovascular Quality and Outcomes*, *10*(11), e004094.

Jardim, T. V., Witham, M. D., Abrahams-Gessel, S., Gómez-Olivé, F. X., Tollman, S., Berkman, L., & Gaziano, T. A. (2018). Cardiovascular Disease Profile of the Oldest Adults in Rural South Africa: Data from the HAALSI Study (Health and Aging in Africa: Longitudinal Studies of INDEPTH Communities). *Journal of the american geriatrics society, 66*(11), 2151–2157. <https://doi.org/10.1111/jgs.15567>

Jennings, E. A., Chinogurei, C., & Adams, L. (2022). Marital experiences and depressive symptoms among older adults in rural South Africa. *SSM-Mental Health*, *2*, 100083.

Jennings, E. A., Farrell, M. T., & Kobayashi, L. C. (2021). Grandchild caregiving and cognitive health among grandparents in rural South Africa. *Journal of aging and health*, *33*(9), 661-673.

Jennings, E. A., Mkhwanazi, N., & Berkman, L. (2020). Receipt of emotional support among rural South African adults. *Ageing & Society*, *40*(5), 1039-1063.

Kämpfen, F., Kohler, I. V., Bountogo, M., Mwera, J., Kohler, H. P., & Maurer, J. (2020). Using grip strength to compute physical health-adjusted old age dependency ratios. *SSM-population health*, *11*, 100579.

Kimuna, S. R., & Makiwane, M. (2007). Older people as resources in South Africa: Mpumalanga households. *Journal of aging and social policy, 19*(1), 97–114. <https://doi.org/10.1300/J031v19n01_06>

Kobayashi, L. C., Glymour, M. M., Kahn, K., Payne, C. F., Wagner, R. G., Montana, L., ... & Berkman, L. F. (2017). Childhood deprivation and later-life cognitive function in a population-based study of older rural South Africans. *Social Science & Medicine*, *190*, 20-28.

Kobayashi, L. C., Mateen, F. J., Montana, L., Wagner, R. G., Kahn, K., Tollman, S. M., & Berkman, L. F. (2019a). Cognitive function and impairment in older, rural south african adults: Evidence from “health and aging in Africa: A longitudinal study of an INDEPTH Community in Rural South Africa.” *Neuroepidemiology, 52*(1–2), 32–40. <https://doi.org/10.1159/000493483>

Kobayashi, L. C., Mateen, F. J., Montana, L., Wagner, R. G., Kahn, K., Tollman, S. M., & Berkman, L. F. (2019b). Socioeconomic gradients in chronic disease risk behaviors in a population-based study of older adults in rural South Africa.” *International journal of public health, 52*(1–2), 32–40. <https://doi.org/10.1159/000493483>

Kobayashi, L. C., Berkman, L. F., Wagner, R. G., Kahn, K., Tollman, S., & Subramanian, S. V. (2019c). Education modifies the relationship between height and cognitive function in a cross-sectional population-based study of older adults in Rural South Africa. *European journal of epidemiology*, *34*, 131-139.

Kobayashi, L. C., Farrell, M. T., Payne, C. F., Mall, S., Montana, L., Wagner, R. G., ... & Berkman, L. F. (2020). Adverse childhood experiences and domain-specific cognitive function in a population-based study of older adults in rural South Africa. *Psychology and aging*, *35*(6), 818.

Kobayashi, L. C., Farrell, M. T., Langa, K. M., Mahlalela, N., Wagner, R. G., & Berkman, L. F. (2021). Incidence of cognitive impairment during aging in rural South Africa: evidence from HAALSI, 2014 to 2019. *Neuroepidemiology*, *55*(2), 100-108.

Kunna, R., San Sebastian, M., & Stewart Williams, J. (2017). Measurement and decomposition of socioeconomic inequality in single and multimorbidity in older adults in China and Ghana: results from the WHO study on global AGEing and adult health (SAGE). *International journal for equity in health*, *16*, 1-17.

Kuteesa, M. O., Wright, S., Seeley, J., Mugisha, J., Kinyanda, E., Kakembo, F., Mwesigwa, R., & Scholten, F. (2014). Experiences of HIV-related stigma among HIV-positive older persons in Uganda – a mixed methods analysis. *Sahara J*, *11*(1), 126–137. <https://doi.org/10.1080/17290376.2014.938103>

Kuuire, Vincent Z., Eric Y. Tenkorang, Prince M. Amegbor, and Mark Rosenberg. "Understanding unmet health-care need among older Ghanaians: a gendered analysis." *Ageing & Society* 41, no. 8 (2021): 1748-1769.

Kyobutungi, C., Egondi, T., & Ezeh, A. (2010). The health and well-being of older people in Nairobi’s slums. *Global health action, 3*(1), 2138. <https://doi.org/10.3402/gha.v3i0.2138>

Lartey, S. T., Magnussen, C. G., Si, L., Boateng, G. O., de Graaff, B., Biritwum, R. B., Minicuci, N., Kowal, P., Blizzard, L., & Palmer, A. J. (2019). Rapidly increasing prevalence of overweight and obesity in older Ghanaian adults from 2007-2015: Evidence from Who-Sage waves 1 & 2. *PLoS one, 14*(8). <https://doi.org/10.1371/journal.pone.0215045>

Lasisi, A. O., & Gureje, O. (2014). Prevalence and correlates of dizziness in the Ibadan Study of Ageing. *Ear, nose, & throat journal, 93*(4-5), E37–E44.

Lasisi, A. O., Abiona, T., & Gureje, O. (2010). Tinnitus in the elderly: Profile, correlates, and impact in the Nigerian study of ageing. *Otolaryngology - head and neck surgery, 143*(4), 510–515. <https://doi.org/10.1016/j.otohns.2010.06.817>

Lee, J. A., & Schafer, M. H. (2023). Social network characteristics and HIV testing among older adults in South Africa. *Ageing & Society*, *43*(3), 499-515.

Lloyd-Sherlock, P., Agrawal, S., & Gómez-Olivé, F. X. (2020). Pensions, consumption and health: evidence from rural South Africa. *BMC Public Health*, *20*(1), 1-10.

Macia, E., Duboz, P., Montepare, J. M., & Gueye, L. (2015). Exploring Life Satisfaction Among Older Adults in Dakar. *Journal of cross-cultural gerontology, 30*(4), 377–391. <https://doi.org/10.1007/s10823-015-9275-8>

Manne-Goehler, J., Montana, L., Gómez-Olivé, F. X., Rohr, J., Harling, G., Wagner, R. G., ... & Gaziano, T. A. (2017). The ART advantage: healthcare utilization for diabetes and hypertension in rural South Africa. *Journal of acquired immune deficiency syndromes (1999)*, *75*(5), 561..

Manne-Goehler, J., Kakuhikire, B., Abaasabyoona, S., Bärnighausen, T. W., Okello, S., Tsai, A. C., & Siedner, M. J. (2019). Depressive symptoms before and after antiretroviral therapy initiation among older-aged individuals in rural Uganda. *AIDS and Behavior*, *23*, 564-571.

Marcus, M. E., Mahlalela, N., Drame, N. D., Rohr, J. K., Vollmer, S., Tollman, S., ... & Bärnighausen, T. (2021). Home-Based HIV Testing Strategies for Older Adults in Rural South Africa: A Randomized Controlled Trial. *Available at SSRN 4090408*.

Martinez, P., Lien, L., Landheim, A., Kowal, P., & Clausen, T. (2014). Quality of life and social engagement of alcohol abstainers and users among older adults in South Africa. *BMC public health, 14*(1), 316. <https://doi.org/10.1186/1471-2458-14-316>

Maritz, M., Fourie, C. M., Van Rooyen, J. M., & Schutte, A. E. (2018). Evaluating several biomarkers as predictors of aortic stiffness in young and older Africans, not consuming alcohol based on self-report. *Diabetes Research and Clinical Practice*, *142*, 312-320.

Matlho, K., Randell, M., Lebelonyane, R., Kefas, J., Driscoll, T., & Negin, J. (2019). HIV prevalence and related behaviours of older people in Botswana—secondary analysis of the Botswana AIDS Impact Survey (BAIS) IV. *African Journal of AIDS Research*, *18*(1), 18-26.

McKinnon, B., Harper, S., & Moore, S. (2013). The relationship of living arrangements and depressive symptoms among older adults in sub-Saharan Africa. *BMC public health, 13*(1), 682. <https://doi.org/10.1186/1471>

Menyanu, E., Charlton, K. E., Ware, L. J., Russell, J., Biritwum, R., & Kowal, P. (2017). Salt use behaviours of Ghanaians and South Africans: a comparative study of knowledge, attitudes and practices. *Nutrients*, *9*(9), 939.

Minicuci, N., Biritwum, R. B., Mensah, G., Yawson, A. E., Naidoo, N., Chatterji, S., & Kowal, P. (2014). Sociodemographic and socioeconomic patterns of chronic non-communicable disease among the older adult population in Ghana. *Global health action, 7*(1). <https://doi.org/10.3402/gha.v7.21292>

Moore, A. R., Prybutok, V., Ta, A., & Amey, F. (2018). Personal social networks and health among aging adults in Agincourt, South Africa: A multidimensional approach. *Social Networks*, *55*, 142-148.

Moreno-Agostino, D., Stone, A. A., Schneider, S., Koskinen, S., Leonardi, M., Naidoo, N., ... & Chatterji, S. (2020). Are retired people higher in experiential wellbeing than working older adults? A time use approach. *Emotion*, *20*(8), 1411.

Mtowa, A., Gerritsen, A. A. M., Mtenga, S., Mwangome, M., & Geubbels, E. (2017). Socio-demographic inequalities in HIV testing behaviour and HIV prevalence among older adults in rural Tanzania, 2013. *AIDS Care, 29*(9), 1162–1168. <https://doi.org/10.1080/09540121.2017.1308462>

Mugisha, J. O., Schatz, E. J., Randell, M., Kuteesa, M., Kowal, P., Negin, J., & Seeley, J. (2016). Chronic disease, risk factors and disability in adults aged 50 and above living with and without HIV: findings from the Wellbeing of Older People Study in Uganda. *Global health action, 9*(1). <https://doi.org/10.3402/gha.v9.31098>

Mugisha, J. O., Schatz, E. J., Negin, J., Mwaniki, P., Kowal, P., & Seeley, J. (2017). Timing of most recent health care visit by older people living with and without HIV: findings from the SAGE well-being of older people study in Uganda. *The International Journal of Aging and Human Development*, *85*(1), 18-32.

Mugisha, J. O., Schatz, E. J., Hansen, C., Leary, E., Negin, J., Kowal, P., & Seeley, J. (2018). Social engagement and survival in people aged 50 years and over living with HIV and without HIV in Uganda: A prospective cohort study. *African Journal of AIDS Research*, *17*(4), 333-340.

Mugisha Okello, J., Nash, S., Kowal, P., Naidoo, N., Chatterji, S., Boerma, T., & Seeley, J. (2020). Survival of people aged 50 years and older by HIV and HIV treatment status: findings from three waves of the SAGE-Wellbeing of Older People Study (SAGE-WOPS) in Uganda. *AIDS Research and Therapy*, *17*, 1-8.

Mugisha, J. O., Schatz, E., Seeley, J., & Kowal, P. (2015). Gender perspectives in care provision and care receipt among older people infected and affected by HIV in Uganda. *African Journal of AIDS Research*, *14*(2), 159-167.

Mwanyangala, M., Mayombana, C., Urassa, H., Charles, J., Mahutanga, C., Abdullah, S., & Nathan, R. (2010). Health status and quality of life among older adults in rural Tanzania. *Global health action*, *3*(1), 2142.

Myroniuk, T. W. (2017). Marital dissolutions and the health of older individuals in a rural African context. *Journals of Gerontology Series B: Psychological Sciences and Social Sciences*, *72*(4), 656-664.

Negin, J., Wariero, J., Cumming, R. G., Mutuo, P., & Pronyk, P. M. (2010). High rates of AIDS-related mortality among older adults in rural Kenya. *JAIDS Journal of Acquired Immune Deficiency Syndromes*, *55*(2), 239-244.

Negin, J., Randell, M., Raban, M. Z., Nyirenda, M., Kalula, S., Madurai, L., & Kowal, P. (2017). Health expenditure and catastrophic spending among older adults living with HIV. *Global public health*, *12*(10), 1282-1296.

Negin, J., Geddes, L., Brennan-Ing, M., Kuteesa, M., Karpiak, S., & Seeley, J. (2016). Sexual behavior of older adults living with HIV in Uganda. *Archives of sexual behavior*, *45*, 441-449.

Negin, J., Martiniuk, A., Cumming, R. G., Naidoo, N., Phaswana-Mafuya, N., Madurai, L., ... & Kowal, P. (2012a). Prevalence of HIV and chronic comorbidities among older adults. *AIDS (london, England)*, *26*(0 1), S55.

Negin, J., Nemser, B., Cumming, R., Lelerai, E., Ben Amor, Y., & Pronyk, P. (2012b). HIV attitudes, awareness and testing among older adults in Africa. *AIDS and Behavior*, *16*, 63-68.

Negin, J., Cumming, R., de Ramirez, S. S., Abimbola, S., & Sachs, S. E. (2011). Risk factors for non‐communicable diseases among older adults in rural Africa. *Tropical Medicine & International Health*, *16*(5), 640-646.

Nikolov, P., Jimi, N., & Chang, J. (2020). The importance of cognitive domains and the returns to schooling in South Africa: Evidence from two labor surveys. *Labour economics*, *65*, 101849.

Nwakasi, C. C., Brown, J. S., & Anyanwu, P. (2019). What could be influencing older Ghanaians outpatient care utilization rate?. *Ghana Medical Journal*, *53*(3), 217-225.

Nyirenda, M., Chatterji, S., Falkingham, J., Mutevedzi, P., Hosegood, V., Evandrou, M., ... & Newell, M. L. (2012). An investigation of factors associated with the health and well-being of HIV-infected or HIV-affected older people in rural South Africa. *BMC Public Health*, *12*, 1-14.

Nyirenda, M., Newell, M. L., Mugisha, J., Mutevedzi, P. C., Seeley, J., Scholten, F., & Kowal, P. (2013). Health, wellbeing, and disability among older people infected or affected by HIV in Uganda and South Africa. *Global health action, 6*(1). <https://doi.org/10.3402/gha.v6i0.19201>

Ogunniyi, A., Hall, K. S., Gureje, O., Baiyewu, O., Gao, S., Unverzagt, F. W., ... & Hendrie, H. C. (2006). Risk factors for incident Alzheimer's disease in African Americans and Yoruba. *Metabolic Brain Disease*, *21*, 224-229.

Ojagbemi, A., Oladeji, B., Abiona, T., & Gureje, O. (2013). Suicidal behaviour in old age-results from the Ibadan Study of Ageing. *BMC psychiatry*, *13*(1), 1-7.

Ojagbemi, A., Abiona, T., Luo, Z., & Gureje, O. (2018). Symptomatic and functional recovery from major depressive disorder in the Ibadan study of ageing. *The American Journal of Geriatric Psychiatry*, *26*(6), 657-666.

Ojagbemi, A., D’Este, C., Verdes, E., Chatterji, S., & Gureje, O. (2015). Gait speed and cognitive decline over 2 years in the Ibadan study of aging. *Gait & posture*, *41*(2), 736-740.

Ojagbemi, A., Bello, T., & Gureje, O. (2016). Cognitive reserve, incident dementia, and associated mortality in the ibadan study of ageing. *Journal of the American Geriatrics Society*, *64*(3), 590-595.

Ojagbemi, A., Bello, T., Luo, Z., & Gureje, O. (2017a). Chronic conditions, new onset, and persistent disability in the Ibadan study of aging. *Journals of Gerontology Series A: Biomedical Sciences and Medical Sciences*, *72*(7), 997-1005.

Ojagbemi, A., Bello, T., Luo, Z., & Gureje, O. (2017b). Living conditions, low socioeconomic position, and mortality in the Ibadan study of aging. *Journals of Gerontology Series B: Psychological Sciences and Social Sciences*, *72*(4), 646-655.

Oladeji, B. D., Makanjuola, V. A., Esan, O. B., & Gureje, O. (2011). Chronic pain conditions and depression in the Ibadan Study of Ageing. *International psychogeriatrics*, *23*(6), 923-929.

Payne, C. F., Mkandawire, J., & Kohler, H. P. (2013). Disability transitions and health expectancies among adults 45 years and older in Malawi: a cohort-based model. *PLoS medicine*, *10*(5), e1001435.

Payne, C. F., Wade, A., Kabudula, C. W., Davies, J. I., Chang, A. Y., Gomez-Olive, F. X., ... & Witham, M. D. (2017a). Prevalence and correlates of frailty in an older rural African population: findings from the HAALSI cohort study. *BMC geriatrics*, *17*(1), 1-10.

Payne, C. F., Gómez-Olivé, F. X., Kahn, K., & Berkman, L. (2017b). Physical function in an aging population in rural South Africa: Findings from HAALSI and cross-national comparisons with HRS sister studies. *The Journals of Gerontology: Series B*, *72*(4), 665-679.

Payne, C. F., Mall, S., Kobayashi, L., Kahn, K., & Berkman, L. (2020). Life-course trauma and later life mental, physical, and cognitive health in a postapartheid South African population: Findings from the HAALSI study. *Journal of aging and health*, *32*(9), 1244-1257.

Peltzer, K., & Pengpid, S. (2018). Self-reported sleep duration and its correlates with sociodemographics, health behaviours, poor mental health, and chronic conditions in rural persons 40 years and older in South Africa. *International journal of environmental research and public health*, *15*(7), 1357.

Peltzer, K., & Phaswana-Mafuya, N. (2012a). Patient experiences and health system responsiveness among older adults in South Africa. *Global Health Action*, *5*(1), 18545.

Peltzer, K., & Phaswana-Mafuya, N. (2012b). Tobacco use and associated factors in older adults in South Africa. *Journal of Psychology in Africa*, *22*(2), 283-288.

Peltzer, K., & Phaswana-Mafuya, N. (2012c). Fruit and vegetable intake and associated factors in older adults in South Africa. *Global health action*, *5*(1), 18668.

Peltzer, K., & Phaswana-Mafuya, N. (2013a). Depression and associated factors in older adults in South Africa. *Global health action*, *6*(1), 18871.

Peltzer, K., & Phaswana-Mafuya, N. (2013b). Arthritis and associated factors in older adults in South Africa. *Turk J Geriatr*, *16*(4), 389-94.

Peltzer, K., & Phaswana-Mafuya, N. (2014). Breast and cervical cancer screening and associated factors among older adult women in South Africa. *Asian Pacific Journal of Cancer Prevention*, *15*(6), 2473-2476.

Peltzer, K., & Phaswana-Mafuya, N. (2017). Association between visual impairment and low vision and sleep duration and quality among older adults in South Africa. *International journal of environmental research and public health*, *14*(7), 811.

Peltzer, K., & Phaswana-Mafuya, N. (2013c). Hypertension and associated factors in older adults in South Africa: cardiovascular topics. *Cardiovascular journal of Africa*, *24*(3), 66-71.

Peltzer, K. (2017). Differences in sleep duration among four different population groups of older adults in South Africa. *International Journal of Environmental Research and Public Health*, *14*(5), 502.

Pengpid, S., & Peltzer, K. (2018). Prevalence and social and health correlates of insomnia symptoms among middle-and older-age persons in rural South Africa. *Journal of Psychology in Africa*, *28*(6), 472-478.

Pengpid, S., & Peltzer, K. (2019). Sedentary behaviour and 12 sleep problem indicators among middle-aged and elderly adults in South Africa. *International journal of environmental research and public health*, *16*(8), 1422.

Pengpid, S., & Peltzer, K. (2020). Mental morbidity and its associations with socio-behavioural factors and chronic conditions in rural middle-and older-aged adults in South Africa. *Journal of Psychology in Africa*, *30*(3), 257-263.

Phaswana-Mafuya, N., & Peltzer, K. (2018). Racial or ethnic health disparities among older adults in four population groups in South Africa. *Annals of global health*, *84*(1), 7.

Phaswana-Mafuya, N., Peltzer, K., Chirinda, W., Musekiwa, A., & Kose, Z. (2013a). Sociodemographic predictors of multiple non-communicable disease risk factors among older adults in South Africa. *Global health action*, *6*(1), 20680.

Phaswana-Mafuya, N., Peltzer, K., Chirinda, W., Musekiwa, A., Kose, Z., Hoosain, E., ... & Ramlagan, S. (2013b). Self-reported prevalence of chronic non-communicable diseases and associated factors among older adults in South Africa. *Global health action*, *6*(1), 20936.

Putnam, H. W., Jones, R., Rogathi, J., Gray, W. K., Swai, B., Dewhurst, M., ... & Walker, R. W. (2018). Hypertension in a resource‐limited setting: Is it associated with end organ damage in older adults in rural Tanzania?. *The Journal of Clinical Hypertension*, *20*(2), 217-224.

Raji, Y. R., Abiona, T., & Gureje, O. (2017). Awareness of hypertension and its impact on blood pressure control among elderly nigerians: report from the Ibadan study of aging. *The Pan African Medical Journal*, *27*.

Ralston, M., Schatz, E., Menken, J., Gómez-Olivé, F. X., & Tollman, S. (2016). Who benefits—or does not—from South Africa’s old age pension? Evidence from characteristics of rural pensioners and non-pensioners. *International Journal of Environmental Research and Public Health*, *13*(1), 85.

Ralston, M. (2018). The role of older persons’ environment in aging well: quality of life, illness, and community context in South Africa. *The Gerontologist*, *58*(1), 111-120.

Ralston, M., Jennings, E., & Schatz, E. (2022). Who is at risk? Social support, relationship dissolution, and illness in a rural context. *Sociological Inquiry*, *92*(3), 1053-1082

Ramlagan, S., Peltzer, K., & Phaswana-Mafuya, N. (2013). Social capital and health among older adults in South Africa. *BMC geriatrics*, *13*, 1-11.

Ramlagan, S., Peltzer, K., & Phaswana-Mafuya, N. (2014). Hand grip strength and associated factors in non-institutionalised men and women 50 years and older in South Africa. *BMC research notes*, *7*, 1-7.

Randall, S., & Coast, E. (2016). The quality of demographic data on older Africans. *Demographic Research*, *34*, 143-174.

Reiger, S., Jardim, T. V., Abrahams-Gessel, S., Crowther, N. J., Wade, A., Gomez-Olive, F. X., ... & Gaziano, T. A. (2017). Awareness, treatment, and control of dyslipidemia in rural South Africa: The HAALSI (Health and Aging in Africa: A Longitudinal Study of an INDEPTH Community in South Africa) study. *PloS one*, *12*(10), e0187347.

Rishworth, A., Elliott, S. J., & Kangmennaang, J. (2020). Getting old well in sub saharan Africa: Exploring the social and structural drivers of subjective wellbeing among elderly men and women in Uganda. *International Journal of Environmental Research and Public Health*, *17*(7), 2347.

Rohr, J. K., Xavier Gómez‐Olivé, F., Rosenberg, M., Manne‐Goehler, J., Geldsetzer, P., Wagner, R. G., ... & Bärnighausen, T. (2017). Performance of self‐reported HIV status in determining true HIV status among older adults in rural South Africa: a validation study. *Journal of the International AIDS Society*, *20*(1), 21691.

Rohr, J. K., Manne-Goehler, J., Gómez-Olivé, F. X., Wagner, R. G., Rosenberg, M., Geldsetzer, P., ... & Salomon, J. A. (2020). HIV treatment cascade for older adults in rural South Africa. *Sexually transmitted infections*, *96*(4), 271-276.

Rosenberg, M. S., Gómez-Olivé, F. X., Rohr, J. K., Houle, B. C., Kabudula, C. W., Wagner, R. G., ... & Bärnighausen, T. (2017). Sexual behaviors and HIV status: a population-based study among older adults in rural South Africa. *Journal of acquired immune deficiency syndromes (1999)*, *74*(1), e9.

Rosenberg, M. S., Gomez-Olive, F. X., Rohr, J. K., Kahn, K., & Baernighausen, T. W. (2018). Are circumcised men safer sex partners? Findings from the HAALSI cohort in rural South Africa. *Plos one*, *13*(8), e0201445.

Rosenberg, M., Gómez‐Olivé, F. X., Wagner, R. G., Rohr, J., Payne, C. F., Berkman, L., ... & Kobayashi, L. C. (2020). The relationships between cognitive function, literacy and HIV status knowledge among older adults in rural South Africa. *Journal of the International AIDS Society*, *23*(3), e25457.

Rossouw, L., & Smith, A. (2017). A comparable yardstick: adjusting for education bias in South African health system responsiveness ratings. *Health Policy and Planning*, *32*(suppl_3), iii67-iii74.

Saeed, B. I. I., Yawson, A. E., Nguah, S., Agyei-Baffour, P., Emmanuel, N., & Ayesu, E. (2016). Effect of socio-economic factors in utilization of different healthcare services among older adult men and women in Ghana. *BMC Health Services Research*, *16*(1), 1-9.

Samba, H., Guerchet, M., Ndamba-Bandzouzi, B., Kehoua, G., Mbelesso, P., Desormais, I., ... & Lacroix, P. (2019). Ankle Brachial Index (ABI) predicts 2-year mortality risk among older adults in the Republic of Congo: The EPIDEMCA-FU study. *Atherosclerosis*, *286*, 121-127.

Sanuade, O. A., Dodoo, F. N. A., Koram, K., & de-Graft Aikins, A. (2019). Prevalence and correlates of stroke among older adults in Ghana: Evidence from the Study on Global AGEing and adult health (SAGE). *PloS one*, *14*(3), e0212623.

Schafer, M. H., Upenieks, L., & DeMaria, J. (2021). Do older adults with HIV have distinctive personal networks? Stigma, network activation, and the role of disclosure in South Africa. *AIDS and Behavior*, *25*, 1560-1572.

Schatz, E. J. (2007). ``Taking care of my own blood'': Older women's relationships to their households in rural South Africa. *Scandinavian journal of public health*, *35*(69_suppl), 147-154.

Schatz, E., Gómez-Olivé, X., Ralston, M., Menken, J., & Tollman, S. (2012). The impact of pensions on health and wellbeing in rural South Africa: does gender matter?. *Social science & medicine*, *75*(10), 1864-1873.

Schatz, E., Madhavan, S., Collinson, M., Gómez-Olivé, F. X., & Ralston, M. (2015). Dependent or Productive? A New Approach to Understanding the Social Positioning of Older South Africans Through Living Arrangements. *Research on aging, 37*(6), 581–605. <https://doi.org/10.1177/0164027514545976>

Schatz, E., Ralston, M., Madhavan, S., Collinson, M. A., & Gómez-Olivé, F. X. (2018). Living arrangements, disability and gender of older adults among rural South Africa. *Journals of gerontology - series B psychological sciences and social sciences, 73*(6), 1112–1122. <https://doi.org/10.1093/geronb/gbx081>

Scholten, F., Mugisha, J., Seeley, J., Kinyanda, E., Nakubukwa, S., Kowal, P., ... & Grosskurth, H. (2011). Health and functional status among older people with HIV/AIDS in Uganda. *BMC Public Health*, *11*(1), 1-10.

Stringhini, S., Carmeli, C., Jokela, M., Avendaño, M., McCrory, C., d’Errico, A., ... & Kivimäki, M. (2018). Socioeconomic status, non-communicable disease risk factors, and walking speed in older adults: multi-cohort population based study. *bmj*, *360*

Tomaz, S. A., Davies, J. I., Micklesfield, L. K., Wade, A. N., Kahn, K., Tollman, S. M., ... & Witham, M. D. (2020). Self-reported physical activity in middle-aged and older adults in rural South Africa: levels and correlates. *International Journal of Environmental Research and Public Health*, *17*(17), 6325.

van Empel, E., de Vlieg, R. A., Montana, L., Gómez-Olivé, F. X., Kahn, K., Tollman, S., ... & Manne-Goehler, J. (2021). Older adults vastly overestimate both HIV acquisition risk and HIV prevalence in rural South Africa. *Archives of sexual behavior*, *50*, 3257-3276.

Wade, A. N., Crowther, N. J., Abrahams-Gessel, S., Berkman, L., George, J. A., Gómez-Olivé, F. X., ... & Cappola, A. R. (2021a). Concordance between fasting plasma glucose and HbA1c in the diagnosis of diabetes in black South African adults: a cross-sectional study. *BMJ open*, *11*(6), e046060.

Wade, A. N., Payne, C. F., Berkman, L., Chang, A., Gómez-Olivé, F. X., Kabudula, C., ... & Davies, J. (2021b). Multimorbidity and mortality in an older, rural black South African population cohort with high prevalence of HIV findings from the HAALSI Study. *BMJ open*, *11*(9), e047777.

Wagner, R. G., Crowther, N. J., Gómez-Olivé, F. X., Kabudula, C., Kahn, K., Mhembere, M., ... & as members of AWI-Gen and the H3Africa Consortium. (2018). Sociodemographic, socioeconomic, clinical and behavioural predictors of body mass index vary by sex in rural South African adults-findings from the AWI-Gen study. *Global health action*, *11*(sup2), 1549436.

Wallrauch, C., Bärnighausen, T., & Newell, M. L. (2010). HIV prevalence and incidence in people 50 years and older in rural South Africa. *SAMJ: South African Medical Journal*, *100*(12), 812-813.

Ware, L. J., Charlton, K., Schutte, A. E., Cockeran, M., Naidoo, N., & Kowal, P. (2017). Associations between dietary salt, potassium and blood pressure in South African adults: WHO SAGE Wave 2 Salt & Tobacco. *Nutrition, Metabolism and Cardiovascular Diseases*, *27*(9), 784-791.

Waterhouse, P., Van Der Wielen, N., Banda, P. C., & Channon, A. A. (2017). The impact of multi-morbidity on disability among older adults in South Africa: do hypertension and socio-demographic characteristics matter?. *International Journal for Equity in Health*, *16*(1), 1-10.

Watkins, D., Brouwer, E., & Nugent, R. (2014). PT321 Economic consequences of cardiovascular disease in South African households: an analysis of the WHO Study on Global Aging (SAGE). *Global Heart*, *9*(1), e229.

Wilunda, B., Ng, N., & Stewart Williams, J. (2015). Health and ageing in Nairobi’s informal settlements-evidence from the International Network for the Demographic Evaluation of Populations and Their Health (INDEPTH): a cross sectional study. *BMC Public Health*, *15*(1), 1-11.

Yawson, A. E., Baddoo, A., Hagan-Seneadza, N. A., Calys-Tagoe, B., Hewlett, S., Dako-Gyeke, P., ... & Biritwum, R. (2013). Tobacco use in older adults in Ghana: sociodemographic characteristics, health risks and subjective wellbeing. *BMC Public Health*, *13*(1), 1-8.

Yawson, A. E., Ackuaku-Dogbe, E. M., Seneadza, N. A. H., Mensah, G., Minicuci, N., Naidoo, N., ... & Biritwum, R. B. (2014). Self-reported cataracts in older adults in Ghana: sociodemographic and health related factors. *BMC public health*, *14*(1), 1-8.

Yoro-Zohoun, I., Houinato, D., Nubukpo, P., Mbelesso, P., Ndamba-Bandzouzi, B., Lambert, J. C., ... & Guerchet, M. (2021). Apolipoprotein E ϵ4 allele and neuropsychiatric symptoms among older adults in Central Africa (EPIDEMCA study). *International Psychogeriatrics*, *33*(3), 295-306.

Yoro‐Zohoun, I., Nubukpo, P., Houinato, D., Mbelesso, P., Ndamba‐Bandzouzi, B., Clément, J. P., ... & EPIDEMCA Group. (2019). Neuropsychiatric symptoms among older adults living in two countries in Central Africa (EPIDEMCA study). *International Journal of Geriatric Psychiatry*, *34*(1), 169-178.

Zengin, A., Fulford, A. J., Sawo, Y., Jarjou, L. M., Schoenmakers, I., Goldberg, G., ... & Ward, K. A. (2017). The Gambian Bone and Muscle Ageing Study: baseline data from a prospective observational African sub-Saharan study. *Frontiers in endocrinology*, *8*, 219.

Zengin, A., Jarjou, L. M., Prentice, A., Cooper, C., Ebeling, P. R., & Ward, K. A. (2018). The prevalence of sarcopenia and relationships between muscle and bone in ageing West‐African Gambian men and women. *Journal of cachexia, sarcopenia and muscle*, *9*(5), 920-928.
